# Solid-state atomic hydrogen as a broad-spectrum RONS scavenger for accelerated diabetic wound healing

**DOI:** 10.1093/nsr/nwad269

**Published:** 2023-10-16

**Authors:** Man Luo, Qin Wang, Gang Zhao, Wei Jiang, Cici Zeng, Qingao Zhang, Ruyu Yang, Wang Dong, Yunxi Zhao, Guozhen Zhang, Jun Jiang, Yucai Wang, Qing Zhu

**Affiliations:** Key Laboratory of Precision and Intelligent Chemistry, School of Chemistry and Materials Science, University of Science and Technology of China, Hefei230026, China; Department of Radiology, The First Affiliated Hospital of University of Science and Technology of China, Division of Life Sciences and Medicine, University of Science and Technology of China, Hefei230026, China; Key Laboratory of Precision and Intelligent Chemistry, School of Chemistry and Materials Science, University of Science and Technology of China, Hefei230026, China; Department of Radiology, The First Affiliated Hospital of University of Science and Technology of China, Division of Life Sciences and Medicine, University of Science and Technology of China, Hefei230026, China; Department of Radiology, The First Affiliated Hospital of University of Science and Technology of China, Division of Life Sciences and Medicine, University of Science and Technology of China, Hefei230026, China; Department of Radiology, The First Affiliated Hospital of University of Science and Technology of China, Division of Life Sciences and Medicine, University of Science and Technology of China, Hefei230026, China; Key Laboratory of Precision and Intelligent Chemistry, School of Chemistry and Materials Science, University of Science and Technology of China, Hefei230026, China; Department of Radiology, The First Affiliated Hospital of University of Science and Technology of China, Division of Life Sciences and Medicine, University of Science and Technology of China, Hefei230026, China; Shenzhen Senior High School, Shenzhen518040, China; Key Laboratory of Precision and Intelligent Chemistry, School of Chemistry and Materials Science, University of Science and Technology of China, Hefei230026, China; Key Laboratory of Precision and Intelligent Chemistry, School of Chemistry and Materials Science, University of Science and Technology of China, Hefei230026, China; Department of Radiology, The First Affiliated Hospital of University of Science and Technology of China, Division of Life Sciences and Medicine, University of Science and Technology of China, Hefei230026, China; Key Laboratory of Precision and Intelligent Chemistry, School of Chemistry and Materials Science, University of Science and Technology of China, Hefei230026, China; Institute of Intelligent Innovation, Henan Academy of Sciences, Zhengzhou451162, China

**Keywords:** atomic hydrogen, RONS scavenger, inflammatory regulation, diabetic wound healing, hydrogen therapy

## Abstract

Hydrogen therapy shows great promise as a versatile treatment method for diseases associated with the overexpression of reactive oxygen and nitrogen species (RONS). However, developing an advanced hydrogen therapy platform that integrates controllable hydrogen release, efficient RONS elimination, and biodegradability remains a giant technical challenge. In this study, we demonstrate for the first time that the tungsten bronze phase H_0.53_WO_3_ (HWO) is an exceptionally ideal hydrogen carrier, with salient features including temperature-dependent highly-reductive atomic hydrogen release and broad-spectrum RONS scavenging capability distinct from that of molecular hydrogen. Moreover, its unique pH-responsive biodegradability ensures post-therapeutic clearance at pathological sites. Treatment with HWO of diabetic wounds in an animal model indicates that the solid-state atomic H promotes vascular formation by activating M2-type macrophage polarization and anti-inflammatory cytokine production, resulting in acceleration of chronic wound healing. Our findings significantly expand the basic categories of hydrogen therapeutic materials and pave the way for investigating more physical forms of hydrogen species as efficient RONS scavengers for clinical disease treatment.

## Teaser

Atomic hydrogen-containing H_x_WO_3_ represents a novel broad-spectrum scavenger of reactive oxygen and nitrogen species, with controllable atomic hydrogen release and pH-responsive biodegradability that effectively alleviates inflammation and accelerates diabetic wound healing.

## INTRODUCTION

Hydrogen therapy is an emerging and promising approach for the treatment of various diseases, such as cancer, inflammatory bowel disease, Alzheimer's disease, ischemia-reperfusion injuries and chronic diabetic ulcers. These conditions involve elevated levels of reactive oxygen and nitrogen species (RONS) including hydrogen peroxide (H_2_O_2_), superoxide anion (•O_2_^−^), hydroxyl radical (•OH) and peroxynitrite anion (ONOO^−^) [1–5]. Broad-spectrum antioxidants against multiple RONS maintain intracellular redox homeostasis, thereby impeding the development and progression of diseases associated with aggravated oxidative damage [6]. Due to the physiological functions of endogenous molecular H_2_ in metabolism and pathological regulation, it can easily diffuse across cell membranes and penetrate into organelles to scavenge malignant RONS—with the end product being harmless H_2_O. Medical H_2_ poses no potential risk of blood poisoning even at high concentrations, making it a highly competitive bio-safe antioxidant to replace either drugs that may cause side effects, or artificial enzyme mimics that eliminate only certain types of RONS [7–9]. Hydrogen therapy also can be adopted as an adjuvant therapy to augment the curative effect of other current clinical treatments. Recent studies have validated its feasibility to combat COVID-19 infections by reducing the pro-inflammatory cytokine storm, and lowering respiratory tract resistance to relieve dyspnea and hypoxemia [10–12].

Despite encouraging advances, efficacy of existing hydrogen therapies remains inherently stagnant owing to the lack of ideal H_2_ carriers. Non-invasive administration modalities, such as H_2_-containing air, water, and saline are readily absorbed, but H_2_ molecules tend to wander aimlessly in the body's circulatory system and may be depleted before reaching the target lesion. The major challenge now is the development of precise H_2_ delivery systems at the microscopic level to improve therapeutic efficacy. We have scrutinized reports on industrial hydrogen storage materials such as LiBH_4_ and Mg(BH_4_)_2_ [13,14]. Although the hydrogen fraction is high, their direct use in biomedicine is not feasible because H_2_ release often requires high temperature or pressure as a driving force and is accompanied by severe safety threats [15]. Instead, *in-situ* H_2_ generation systems based on chemically active metals/non-metals and their hydrides (e.g. Fe [3], Mg [16,17], CaH_2_ [18], PdH_x_ [19,20], H_x_TiO_2_ [21], SiH_x_ [22], NH_3_BH_3_ [23]) require exogenous stimuli or violent reactions with water and acid. Yet another important but neglected aspect of such methods is the unknown biocompatibility of aforementioned materials, which is a key priority for practical use. In brief, an ideal carrier for hydrogen therapy should manifest all the merits integrating high hydrogen loading, controlled release, efficient and versatile RONS removal, and last but not least, biodegradability. Each of the above is crucial, as the overall curative effect is often determined by the weakest link according to Cannikin's law.

**Figure 1.**
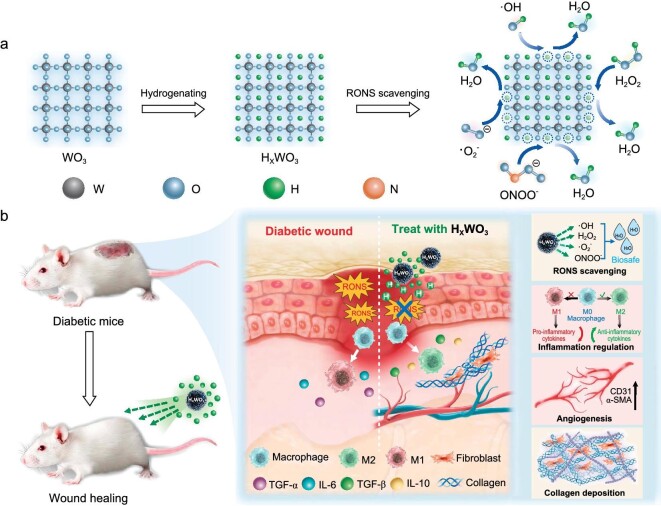
Schematic illustration of solid-state atomic hydrogen as a strong and broad-spectrum RONS scavenger and its therapeutic mechanism in diabetic wound healing. (a) H_x_WO_3_ as an ideal carrier of atomic hydrogen for efficient elimination of RONS. (b) The postulated mechanism of H_x_WO_3_ in accelerating diabetic wound healing, including RONS scavenging, anti-inflammation, angiogenesis and collagen deposition.

The effect of H-doping on the electronic and physicochemical properties of metal oxides has been extensively studied [24,25], but the biological activity of the solid-state atomic H itself for hydrogen therapy has been inadvertently unexplored. From the perspective of thermodynamics and chemical kinetics, the direct use of atomic H is more efficient than H_2_ because of its highly reductive nature (−2.1 V *vs*. RHE) [26], and a smaller size that enhances *in vivo* depth to better scavenge RONS. Unfortunately, converting the H_2_ molecule into an isolated atomic H is extremely difficult due to the high dissociation energy of the H−H bond (*ca*. 436 kJ/mol) [27]. Hence, we aim to improve hydrogen therapeutic efficacy from another starting point, by seeking out alternative carriers capable of directly storing the highly reductive atomic H. One promising candidate is hydrogen tungsten bronze, H_x_WO_3_ (0 < *x* < 1), a solid-state hydrogen carrier in which H atoms can be reversibly inserted and extracted. The use of H_x_WO_3_ as a hybrid electron-proton conductor in electrochromic devices [28,29], hydrogen-transfer catalysts [30,31], H_2_ fuel cells [32,33] and sensors [34,35] has attracted widespread interest, hinting at the feasibility of WO_3_ as an atomic hydrogen carrier. In addition, the biocompatibility of the tungsten-oxygen material system has been widely verified, further avoiding the risk of physiological repulsion and post-treatment toxicity. Herein in this work, we present the first demonstration that hydrogen tungsten bronze (H_0.53_WO_3_) integrates all the key features that an ideal hydrogen therapy platform should possess. The atomic H deposited in H_0.53_WO_3_ showed strong reducibility with temperature-dependent sustainable release behavior and broad-spectrum RONS scavenging capabilities (Fig. 1a). Its distinctive pH-responsive biodegradability also ensured post-therapeutic clearance. Further, we propose that the highly-reductive atomic H can significantly remodel the diabetic wound microenvironment by promoting collagen deposition and reducing inflammation via macrophage M2 polarization. These processes accelerate angiogenesis and ultimately expedite the wound healing process against diabetic ulcers (Fig. 1b). Therefore, it is foreseeable that H_0.53_WO_3_—a biodegradable substance containing the highly reductive atomic hydrogen that serves as an efficient and broad-spectrum RONS scavenger—can regulate inflammation and simultaneously promote the restoration of chronic wounds, thus holding great promise for translation to the clinic.

## RESULTS AND DISCUSSION

### Synthesis and characterization of hydrogen tungsten bronze deposited with atomic hydrogen

**Figure 2.**
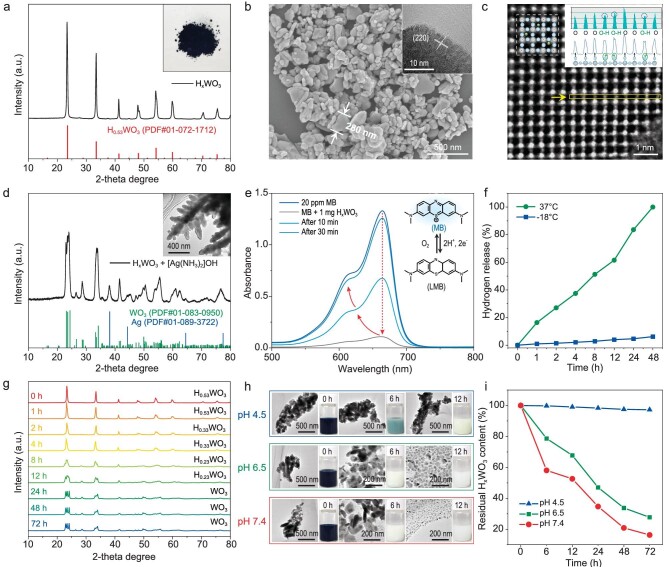
Synthesis and characterization of tungsten bronze phase H_x_WO_3_ containing atomic hydrogen. (a) XRD pattern with standard diffraction pattern of H_0.53_WO_3_ (JCPDS No. 72-1712) beneath the plot. (b) SEM and HRTEM image of H_x_WO_3_. (c) The iDPC-STEM image of H_x_WO_3_ from [010] orientation; the inset is a schematic crystal structure indicated by the yellow arrow. (d) XRD patterns of the precipitate after mixing H_x_WO_3_ and Tollens’ reagent with standard WO_3_ (JCPDS No. 83-0950) and Ag (JCPDS No. 89-3722) as reference. (e) Dynamic evolution of the UV-Vis absorbance spectra of MB probe after atomic hydrogen reduction. (f) Release rate of solid-state atomic hydrogen in H_x_WO_3_ at 37°C and −18°C. (g) The *ex-situ* XRD patterns of H_x_WO_3_ during hydrogen release at 37°C. (h) HRTEM images displaying the collapse of H_x_WO_3_ nanoparticles during the pH-responsive degradation from 0 h to 12 h. (i) The percentage of H_x_WO_3_ residue over time at different pH (4.5, 6.5 and 7.4).

A wet chemical method for hydrogenation of biocompatible WO_3_ was based on the electron-proton co-doping strategy proposed by our group, employing Cu metal and hydrochloric acid to provide electrons and protons, respectively [36–38]. The fabrication process is very facile under ambient conditions and can be scaled up in high yield (Fig. S1a). As time went by, the light-colored WO_3_ gradually converted to dark-black H_x_WO_3_ (Fig. S1b), and H-doping induced topological transformation from trigonal WO_3_ to cubic H_0.53_WO_3_, as confirmed by X-ray diffraction (XRD) (Fig. S1c) [35]. Given that saturated hydrogen-doped H_x_WO_3_ was identified as H_0.53_WO_3_ (denoted HWO) by XRD (Fig. 2a), all subsequent experiments used the HWO serving as the hydrogen therapy platform. As revealed by our density functional theory (DFT) calculations at the PBE + U level (Fig. S2), the reduced Gibbs free energy indicated that the H-doping process was thermodynamically favorable and prone to occur, thus the as-prepared HWO displayed systemic stability. Good hydrophilicity is a prerequisite for dispersion and *in vivo* biosafety of hydrogen carriers; the ^1^H solid-state nuclear magnetic resonance (^1^H-NMR) spectrum (Fig. S3) showed a chemical shift of H dopants appearing in the 1–2 ppm range, along with a surface-adsorbed H_2_O molecule signal at around 6 ppm. The bulging of the background baseline reflects the enhanced electrical conductivity of the material.

The fitted X-ray photoelectron spectroscopy (XPS) spectra in the O *1* *s* region (Fig. S4) produced a tungsten-hydroxyl (W−O−H) like plateau at 531.05 eV in addition to surface-adsorbed H_2_O (532.25 eV), consistent with the NMR results. The existence of W^4+^ and W^5+^ is due to the charge compensation upon H insertion. The incorporation of Cu^2+^ during the hydrogenation of HWO was excluded by XPS spectrum (Fig. S5) [39]. XPS at the valence band disclosed new electron-occupation states near the Fermi level, which facilitates charge transfer in redox-catalyzed reactions. The scanning electron microscopy (SEM) images in Fig. 2b and Fig. S6 demonstrate that the microstructure of WO_3_ is almost unchanged after hydrogenation, implying a mild degree of H-doping. Although the small-sized H dopants caused significant lattice distortion, the particle size remained nearly constant with an average hydrodynamic diameter of 240 nm (Fig. S7), which still meets the essential requirements for non-intravenous biomedical applications. With the most advanced integrated differential phase contrast scanning transmission electron microscopy (iDPC-STEM) that enables the simultaneous resolution of both light and heavy elements, we found that each atomic H is in close proximity to a lattice oxygen, as evidenced by the peak splitting of the line scan profile along the O-chain direction in [40,41].

### Highly-reductive atomic hydrogen exhibits controllable release behavior

Reaction of HWO with Tollens’ reagent ([Ag(NH_3_)_2_]OH) was carried out to verify the chemical reducing power of atomic H. The XRD pattern of the collected precipitate in Fig. 2d showed clear signals of metallic Ag (JCPDS No. 89-3722) that intermingled in a series of characteristic peaks of WO_3_. The reduction of Ag^+^ to Ag^0^ was also supported by the appearance of dendritic Ag nanostructures observed on WO_3_, as visualized by high-resolution transmission electron microscopy (HRTEM) (inset in Fig. 2d). These results imply that HWO eventually reverts back to the original WO_3_ after atomic H oxidation, so that atomic H can be reversibly inserted and extracted without destroying the host lattice [4]. Further, reduction brought about by atomic H is far superior to that of H_2_ molecules, as was indicated by the immediate reduction of methylene blue (MB), a thiazine dye that appears bright blue in its oxidized state and can be reduced to colorless leuco methylene blue (LMB); this reduction can be driven only by atomic H and not by H_2_ [18,20]. As expected, the color of MB solution faded quickly upon adding HWO, as monitored by the dramatic weakening of maximum extinction at 663 nm in its ultraviolet-visible (UV-Vis) spectrum (Fig. 2e). In stark contrast, gaseous H_2_ failed to cause any change in the color of a MB solution (Fig. S8). The fading could be attributed to neither dye adsorption nor degradation because the decolorization caused by HWO was reversible and the LMB obtained could be switched back to MB by exposure to air.

Given the ability of atomic hydrogen in HWO to reduce MB to LMB, the use of MB as a reliable indicator allows for quantitative measurement of the efficiency of atomic hydrogen release. The release rate was clearly temperature-dependent (Fig. 2f), which certainly could improve the convenience and flexibility in biomedical applications. We kept solid HWO at 37°C and −18°C for 1, 2, 4, 8, 12, 24, and 48 hours, and then added it rapidly to an aqueous MB solution (25 ppm). By measuring the corresponding UV-Vis spectral changes based on Beer-Lambert's law (Fig. S9), we quantified the temperature-controlled hydrogen release rate (Fig. S10). Notably, at body temperature (∼37°C), 83.6% of the total atomic H was released within 24 hours and the remaining amount was completely depleted after 2 days (Fig. 2f). This makes it easy to use as no extra exogenous stimuli are needed to exert therapeutic efficacy. In contrast, only 6% hydrogen was released after 48 hours at −18°C, a rate low enough to be well suited for refrigerator storage in daily use. The *ex-situ* XRD patterns (Fig. 2g and Fig. S11) further corroborated the MB titration results of the temperature-controlled release of atomic H in solid HWO.

For therapeutic applications, the biodegradability of hydrogen carriers should also be given priority as it usually relates to whether any residue left after treatment will negatively impact normal tissues. Interestingly, we found that HWO degraded promptly—accompanied by the loss of reducibility—when placed in a pH 7.4 buffer solution. Gradual decomposition of HWO was discerned through HRTEM imaging (Fig. 2h), indicating that this material may be metabolized in a normal physiological environment and does not accumulate in healthy tissues. Notably, the size of HWO can be decomposed to less than 5 nm within 24 hours (Fig. S12). Inductively coupled plasma mass spectrometry (ICP-MS) was utilized to investigate the degradation of WO_3_ and HWO. Remarkably, both materials exhibited pH-responsive degradability (Fig. S13), although HWO dissolved more slowly due to the fact that its H dopant can shield HWO from attack by hydroxyl ions sacrificially. Although under acidic conditions at pH = 4.5, HWO exhibited robustness and barely decomposed at all (Fig. 2i), its degradation rate reached as high as 79.14% within 48 hours at the physiological pH = 7.4, thus conferring appropriate biosafety for hydrogen therapy.

### Broad-spectrum RONS scavenging assays and theoretical evaluation of the atomic hydrogen-mediated therapeutic mechanism

**Figure 3.**
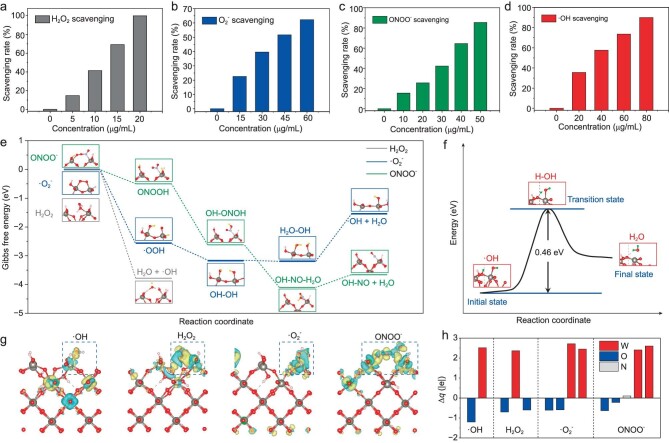
Broad-spectrum RONS scavenging capabilities of atomic hydrogen in H_x_WO_3_ and its theoretical reaction pathways during RONS elimination. The scavenging rate of (a) hydrogen peroxide (H_2_O_2_), (b) superoxide anion (•O_2_^−^), (c) peroxynitrite anion (ONOO^−^), and (d) hydroxyl radicals (•OH) at different H_x_WO_3_ concentrations. (e) Gibbs free energy diagram for each elementary step of reactions between H_x_WO_3_ and H_2_O_2_, •O_2_^−^, and ONOO^−^. (f) Reaction coordinate for surface atomic H combining with chemisorbed •OH to form H_2_O on the H_x_WO_3_ (110) surface as determined by DFT calculations using LASP. (g) Differential charge density of the adsorbed state of •OH, H_2_O_2_, •O_2_^−^, and ONOO^−^ (from left to right). Yellow bubbles represent electron accumulation and cyan, electron depletion. (h) The change in Bader charge (Δ*q*) of W in H_x_WO_3_ and RONS (•OH, H_2_O_2_, •O_2_^−^, and ONOO^−^) as they combined. Negative and positive values indicate reduction and oxidation, respectively.

Different from other hydrogen therapy platforms, the biofunctionality of HWO is based on its ability to react directly with malignant RONS, due to the ability of the more bioreductive atomic H to offer broad-spectrum RONS removal that is not available with H_2_ molecules [2]. To validate this broad-spectrum scavenging capability, we selected four representative RONS (H_2_O_2_, •OH, •O_2_^−^ and ONOO^−^) that are intimately related to the delayed healing of chronic diabetic wounds [41,42]. As shown in Fig. 3a–d, the removal of each type of RONS by HWO proceeded in a concentration-dependent manner, demonstrating its broad-spectrum RONS scavenging ability. Treatment with a low dose of HWO at 20 μg/mL resulted in an impressive >99% H_2_O_2_ clearance (Fig. S14). When the HWO dose was increased to a moderate 60 μg/mL, the removal efficiency of •O_2_^−^ was 63% (Fig. S15). Similarly, HWO exhibited a significant scavenging efficiency of 85% towards the classical RONS species of ONOO^−^ at a concentration of 50 μg/mL (Fig. S16). Scavenging of •OH is crucial because natural organisms lack relevant enzymes; the removal efficiency of the highly cytotoxic •OH reached an excellent 92% at HWO doses of 80 μg/mL (Fig. S17). Compared to previous reports on therapeutic hydrogen carriers or materials with multiple enzymatic activities that could scavenge partial RONS, HWO worked at comparable or even lower concentrations, suggesting its outstanding antioxidant capacity (Table S1).

To gain a deeper insight into the underlying chemical mechanism, density functional theory (DFT) was used to investigate the RONS scavenging pathways of HWO at the atomic scale. Based on the exposed crystal facet of HWO revealed by the HRTEM image (inset in Fig. 2b), we cleaved the (110) surface of bulk H_0.5_WO_3_ as a slab model to simulate the presumptive reaction pathway between HWO and RONS (Fig. S18). The reaction pathway of ONOO^−^ was simulated and determined based on the homolytic cleavage mechanism proposed by Beckman [43] and Radi [44]. Accordingly, ONOO^−^ easily combines with protons to form ONOOH and undergoes the homolytic pathway to produce •OH and •NO_2_, respectively. The calculated Gibbs free energy profile shows that both the adsorption and hydrogenation of H_2_O_2_, •O_2_^−^ and ONOO^−^ proceed spontaneously on the HWO surface (Fig. 3e). Atomic H on the exposed (110) facet of H_0.5_WO_3_ serves as a hydrogen source to reduce the adsorbed RONS via formation of O−H bonds (Fig. S19). Hydrogenation of all RONS species terminates spontaneously in the production of chemisorbed •OH as all reactions up to this step are exothermic, which then forms H_2_O by a combination with nearby surface atomic H (Fig. 3f). The energy barrier (*ΔE*) of hydroxyl radical neutralization reaction was estimated to be 0.46 eV by a first-principles transition state search using the large-scale atomistic simulation program (LASP), implying that the reaction can occur easily under ambient conditions. Besides the promoting role of reactive H atoms, significant interfacial charge transfer from the surface and subsurface W atoms to adsorbed RONS also substantially facilitates their clearance by hydrogenation, as indicated by the differential charge profile (Fig. 3g) and Bader charge distribution (Fig. 3h). Theoretical calculations well explain the strong reduction ability of solid-state atomic H to efficiently cleave the most common RONS, its heightened reactivity comes from the unpaired electrons in the valence electron orbitals to efficiently scavenge a broader range of reactive oxygen and nitrogen species (RONS). It is also foreseeable that our H_x_WO_3_ offers precise control over therapeutic dosing through its temperature-dependent release behavior, presenting apparent advantage over the transient and focused release of H_2_ gas. Our study introduces an innovative paradigm in hydrogen therapy by harnessing the remarkable reactivity of atomic hydrogen to further explore its potential for *in vivo* biomedical applications associated with oxidative damage and exacerbated inflammation caused by RONS overexpression.

### Promotion of diabetic wound healing by atomic hydrogen-induced collagen deposition and angiogenesis

Recent studies support the notion that oxidative stress caused by excess RONS plays a key role in deterioration of chronic diabetic wounds by driving persistent expression of proinflammatory cytokines, which cause oxidative damage and lead to extracellular matrix (ECM) destruction [22,45]. In light of the efficient and broad-spectrum RONS scavenging capability of atomic H in HWO, we therefore evaluated its potential impact on diabetic wound closure efficacy in a diabetic mouse model. The biocompatibility of HWO was first pre-assessed by measurement of serum biochemical parameters and histological analysis of major organs (including heart, liver, spleen, lungs and kidneys) to examine its feasibility for *in vivo* administration. Hematoxylin and eosin (H&E) staining of organ tissues at days 3 and 7 post HWO treatment showed no perceivable histopathological structure abnormalities among treatment groups (Fig. S20), suggesting that HWO did not cause any damage to these organs. Values of all routine blood parameters were also normal (Fig. S21).

The degradation of HWO is crucial to ensure its long-term biosafety; otherwise, nondegradable materials might persist *in vivo* for an extended period after wound healing, posing potential toxicity. Biodegradability of HWO was evaluated by the detection of W contents in the wound tissue at days 3 and 7 using ICP-MS (Fig. S22). The levels of HWO residue in wound areas gradually decreased over time and approached 0% after one week, indicating almost complete HWO degradation, consistent with the results of *in vitro* experiments. These data illustrate that HWO usage did not induce apparent local or systemic cytotoxicity in mice.

**Figure 4.**
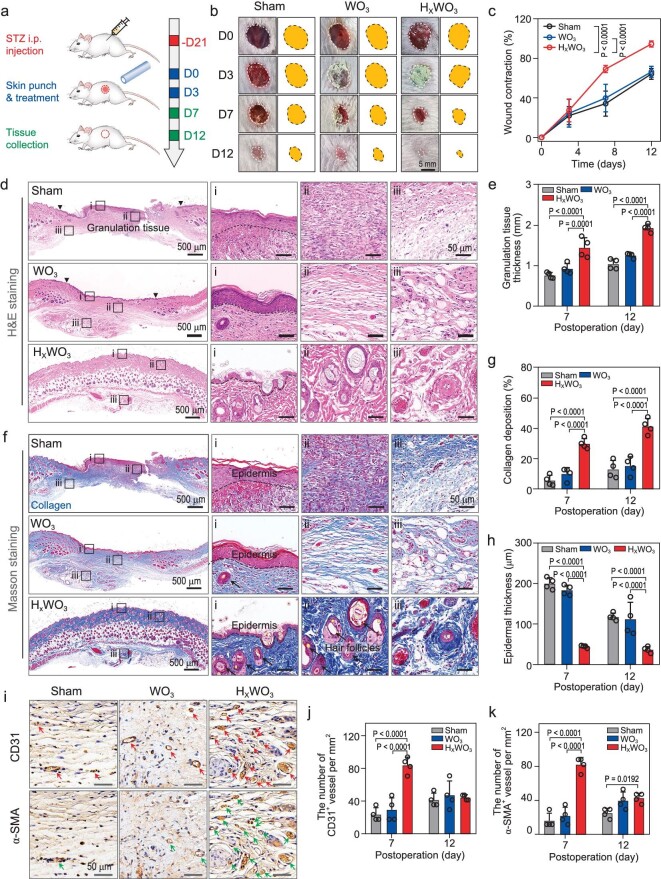
Therapeutic efficacy of chronic wound healing by atomic hydrogen in H_x_WO_3_. (a) Schematic design of the diabetic wound model in the SD mouse. (b) Representative images of the dynamic wound healing process on day 0, 3, 7, and 12 treated with indicated materials. (c) Wound contraction rate on day 0, 3, 7, and 12 (*n* = 4 mice per group). (d) H&E staining images of wound tissue on day 12, accompanied by local magnification of the epidermis (і), dermis (ii) and subcutaneous tissue (iii). (e) Quantification of granulation tissue thickness on days 7 and 12 (*n* = 4 mice per group). (f) Masson's trichrome staining of the wound tissue for visualizing collagen distribution (blue) on day 12, accompanied by local magnification of the epidermis (і), dermis (ii) and subcutaneous tissue (iii). Quantification of (g) collagen deposition and (h) epidermal thickness of wound tissue on days 7 and 12 (*n* = 4 mice per group). (i) Representative images of CD31 and α-SMA immunostaining in wound tissue at day 7. Quantification of the number of (j) CD31^+^ and (k) α-SMA^+^ vessels in wound tissue (*n* = 4 mice per group). Data are shown as the mean ± s.d. Statistical significance was determined using two-way ANOVA with Sidak's multiple comparisons test.

On this basis, a diabetic mouse model with diabetes induced by streptozotocin (STZ) was constructed and used to investigate diabetic wound healing (Fig. 4a). The fasting blood glucose level of mice exceeded 16.7 mmol/L for three consecutive days after STZ treatment, indicating that the diabetic model was successfully established (Fig. S23). Then, wounds produced by a dermatome were treated either with pristine WO_3_ or with HWO. As expected, the contraction rate following HWO treatment was up to 95% at day 12 (Fig. 4b), indicating achievement of an excellent therapeutic effect. During the dynamic wound healing process (Fig. 4c), the wound area after HWO treatment exhibited significantly greater contraction compared with a hydrogen-free WO_3_ counterpart (∼66%) and the group without any treatment (∼64%).

The emergence of fresh granulation tissue and angiogenesis is of great importance for wound repair and regeneration [46,47]. Hence, histology at day 7 and 12 post-wounding was assessed to further investigate the promoting mechanism of wound healing by atomic H. Hematoxylin and eosin (H&E) staining of wound tissue was performed and revealed an obvious increase in thickness of granulation tissue of injured skin covered by HWO (Fig. 4d and e). Moreover, Masson staining allowed for the observation of apparent collagen deposition following HWO treatment (Fig. 4f and g), as well as abundant hair follicle and sebaceous gland formation. In addition to an excessive delay in wound healing, excessive regeneration that may lead to scar formation should also be avoided. Notably, the epidermal thickness in the HWO group was the thinnest, denoting that the entire process from proliferation to remolding had been accelerated moderately by atomic H (Fig. 4h and Fig. S24).

To explore the effect of HWO on angiogenesis, immunofluorescence staining of CD31 and α-smooth muscle actin (α-SMA) was performed for histological analysis. As shown in Fig. 4i, an evidently higher density of vessels was observed in the HWO groups on day 7 than that of the blank group and WO_3_. Remarkably, the number of vessels reached ∼80 per mm^2^, with capillaries throughout the HWO-treated diabetic wound at day 7 (Fig. 4j and k); Furthermore, the vessel programmed death that occurred after 12 days left behind larger vessels with an average diameter of 30 μm (Figs S25 and S26). Collectively, these results indicate that atomic H was intimately involved in the physiological process of diabetic wound healing by effectively promoting the regeneration of granulation tissue, angiogenesis, and collagen deposition in the vicinity of the wound area.

### Effects of atomic hydrogen on inflammatory regulation *in vivo*

**Figure 5.**
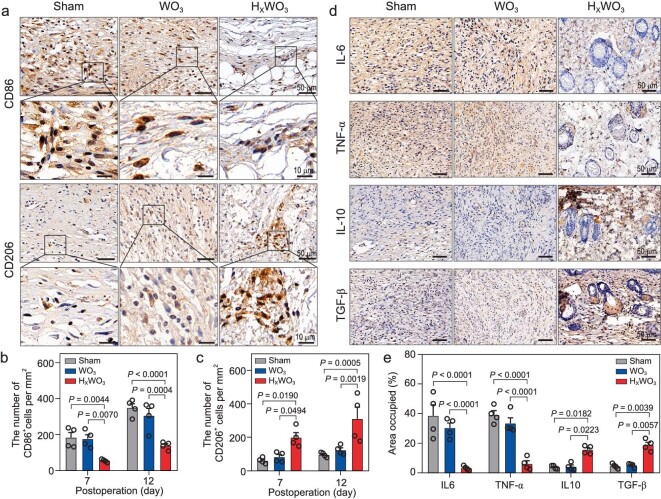
Modulation of macrophage polarization and inflammatory cytokine levels by atomic hydrogen in H_x_WO_3_*in vivo*. (a) Representative images of CD86 and CD206 immunostaining in wound tissue on day 12. Number of (b) CD86^+^ cells and (c) CD206^+^ cells per mm^2^ (*n* = 4 mice per group). (d) Representative images of IL-6, TNF-α, IL-10, and TGF-β staining in wound tissue on day 12. (e) Quantification of cytokine area in wound tissue on day 12 (*n* = 4 mice per group). Data are shown as the mean ± s.d. Statistical significance was determined using two-way ANOVA with Sidak's multiple comparisons test.

In the immediate aftermath of tissue injury, multiple biological pathways are activated and respond in parallel [48]. The inflammatory environment of a diabetic wound slows down the healing process. In particular, macrophages are thought to be essential for coordinating later events in response to wound healing, and regulate the expression of inflammatory cytokines by differentiating into the proinflammatory M1 phenotype or anti-inflammatory M2 phenotype. However, diabetes causes a dysfunctional macrophage response and impaired phenotypic transition from M1 to M2. Exposure of wounds to HWO containing atomic H significantly intensified the polarization of macrophages toward the M2 phenotype on days 7 and 12, as clearly identified by representative immunohistochemistry staining assays of CD86 (M1 marker) and CD206 (M2 marker) (Fig. 5a and Fig. S27). A considerable decline in the number of M1 macrophages (CD86^+^) accompanied by a progressive increase in the proportion of M2 macrophages (CD206^+^) was observed in HWO-treated wounds, whereas the trend was exactly the opposite in the other groups. It was also found that the number of M2 macrophages was greater than that of M1 macrophages in the HWO-cured wounds, while untreated or WO_3_-treated diabetic skin wounds were dominated by M1 macrophages (Fig. 5b and c).

Apart from the phenotype of macrophages, proinflammatory cytokines secreted at the wound location are also an important indicator of the inflammation response. As shown in Fig. 5d, secretion of proinflammatory interleukin-6 (IL-6) and tumor necrosis factor–α (TNF-α) was high in the untreated and WO_3_-treated wounds, demonstrating the presence of excessive inflammatory cells. In contrast, HWO treatment reduced the levels of these proinflammatory factors in diabetic wounds, decreasing IL-6 expression by ∼92% and TNF-α expression by ∼84% (Fig. 5e). Moreover, this treatment can efficiently eliminate RONS and lead to high-level expression of the anti-inflammatory cytokines interleukin 10 (IL-10) and transforming growth factor–β (TGF-β). These findings provide solid evidence of the promising potential of HWO for regulating chronic cutaneous inflammation by transdermal release of atomic H and serving as an inhibitor of oxidative stress.

## CONCLUSIONS

In summary, we demonstrated the major advantages of atomic hydrogen in tungsten bronze phase H_0.53_WO_3_ over conventional H_2_ gas as a hydrogen-centric therapeutic platform for RONS overexpression-related diabetic wound healing. HWO perfectly combines atomic H loading, controllable release, highly effective broad-spectrum RONS scavenging that is superior to any previously reported hydrogen therapeutic materials. Furthermore, the large-scale production of cost-effective HWO under mild conditions offers significant advantages in terms of fabrication accessibility. The activation of the atomic hydrogen stored in HWO is solely temperature-dependent, which completely eliminates the need for external stimuli. And its direct use on the wound surface does not require the involvement of *in vivo* delivery, thus greatly enhancing user-friendliness. Pre-assessment of pH-responsive biodegradable HWO for *in vivo* administration has demonstrated its inherent biosafety. Theoretical and experimental results reveal that solid-state atomic H can convert RONS directly into water with high efficiency. In a diabetic animal model, HWO treatment showed unique positive effects of atomic H on regulating the expression of favorable anti-inflammatory cytokines, on stimulating cell proliferation and angiogenesis, thus on accelerating chronic diabetic wound healing. Our study provides valuable insights that greatly expand the research scope of hydrogen therapy, and thus advance this promising therapeutic approach toward clinical applications.

## METHODS

The details about the synthesis, characterizations and biological performances of HWO are in the Supplementary data.

## Supplementary Material

nwad269_Supplemental_File
