# Supplementary material for: Solid-state atomic hydrogen as a broad-spectrum RONS scavenger for accelerated diabetic wound healing
Source: Natl Sci Rev. 2023 Oct 16;11(2):nwad269. doi: 10.1093/nsr/nwad269 (PMC10776359; doi:10.1093/nsr/nwad269)
Supplement: nwad269_Supplemental_File [file nwad269_supplemental_file.pdf]

-

Supporting Information for

Solid-State Atomic Hydrogen as a Broad-Spectrum RONS Scavenger for Accelerated Diabetic Wound Healing

Man Luo[1,†], Qin Wang[2,†], Gang Zhao[1,†], Wei Jiang[2], Cici Zeng[2], Qingao Zhang[2], Ruyu Yang[1], Wang Dong[2], Yunxi Zhao[3], Guozhen Zhang[1], Jun Jiang[1], Yucai Wang[2,*] and Qing Zhu[1,*]

[1]Key Laboratory of Precision and Intelligent Chemistry, School of Chemistry and Materials Science, University of Science and Technology of China, Hefei 230026, China;
[2]Department of Radiology, The First Affiliated Hospital of University of Science and Technology of China, Division of Life Sciences and Medicine, University of Science and Technology of China, Hefei 230026, China;
[3]Shenzhen Senior High School, Shenzhen 518040, China

*__Corresponding authors.__ E-mails: yucaiwang@ustc.edu.cn; qingzhu@ustc.edu.cn
†Equally contributed to this work.

-

METHODS

Materials

Pristine WO_3 (< 200 nm, Catalog No. T103856-5g), ferrous sulfate ($FeSO_4$), ceric sulfate ($Ce(SO_4)_2$), disodium edetate dihydrate (EDTA-2Na), diethylene triamine pentaacetic acid (DTPA) and Evans Blue (EB) were obtained from Aladdin Reagent (Shanghai, China). Metal copper foil, manganese dioxide (MnO_2), hydrochloric acid (37%), hydrogen peroxide (H_2O_2), potassium nitrite (KNO_2), silver nitrate ($AgNO_3$), ammonia monohydrate ($NH_3·H_2O$), sodium hydroxide (NaOH), sodium chloride (NaCl), potassium chloride (KCl), acetic acid (CH_3COOH) and sodium acetate trihydrate ($C_2H_9NaO_5$) were obtained from China National Pharmaceutical Group Co., Ltd. Methylene Blue (MB), salicylic acid (SA), pyrogallol ($C_6H_3(OH)_3$), tris(hydroxymethyl)aminomethane hydrochloride (Tris-HCl), and streptozotocin (STZ) were purchased from Macklin Biochemical Technology Co., Ltd (Shanghai, China). All chemical reagents were received and used directly without any further treatment. Deionized water (18.2 MΩ/cm) was available using a Milli-Q purification system (Millipore, Bedford, MA, USA).

Synthesis of H_xWO_3

Hydrogen tungsten bronze (H_xWO_3) was facilely synthesized on large scale via a wet-chemical method. Typically, commercial WO_3 nanoparticles (0.1 g) were thoroughly dispersed in an aqueous hydrochloric acid solution (40 mL, 4 M) by slight ultrasonication, then copper metal foil (0.15 g) was added under constant stirring (800 rpm) for 10 h to obtain fully hydrogenated $H_{0.53}WO_3$. The final black product was centrifuged and washed with deionized water and ethanol three times to remove adsorbed impurity ions, and then dried at 60°C under vacuum for further characterization. The synthetic process does not lose any chemical components, thus the yield of as-prepared H_xWO_3 is close to 100%.

pH-responsive degradation of H_xWO_3 *in vitro*

Degradation of H_xWO_3 incubated in phosphate buffer (~6.5 and 7.4) and acetate buffer (~4.5) at different pH values was measured by HRTEM and ICP-MS over time to investigate the morphological changes and metal leaching in the buffer solution.

Measurement of hydroxyl radical (·OH) scavenging activity

Hydroxyl radical scavenging activity was measured using SA as probe, which can be colored by hydroxyl radicals generated from the decomposition of hydrogen peroxide. Typically, 1 mL $FeSO_4$ (3 mM), 1 mL H_2O_2 (1 mM), $H_{0.53}WO_3$ (0, 20, 40, 60 or 80 µg/mL) and 1 mL SA (10 mM) were added to water. After 15 minutes of incubation, the light absorbance at 527 nm was recorded.

Measurement of hydrogen peroxide (H_2O_2) scavenging activity

Hydrogen peroxide scavenging activity was measured using $Ce(SO_4)_2$, which is bleached by H_2O_2. Specifically, H_2O_2 (0.1 mM), $H_{0.53}WO_3$ (0, 5, 10, 15 or 20 µg/mL) and $Ce(SO_4)_2$ (0.5 mM) were added to water. After 15 minutes of incubation, the light absorbance at 319 nm was recorded.

Measurement of superoxide anion ($·O_2^-$) scavenging activity

Superoxide anion scavenging activity was measured by pyrogallol, which is rapidly oxidized under alkaline conditions to release superoxide anions and generate stable-colored intermediates. Firstly, 10 mL of EDTA-2Na (5 mM) was added to 40 mL of Tris-HCl solution (0.5 mM), and NaOH solution (1 M) was added dropwise to adjust the pH~7.4 to obtain solution A. Secondly, pyrogallol was dissolved in HCl (1 mM) to obtain a concentration of 60 mM and marked as solution B. Next, solution A was added to solution B to further

dilute the pyrogallol to a final concentration of 10 mM (labelled as solution C). Lastly, $H_{0.53}WO_3$ (0, 15, 30, 45 or 60 μg/mL) was added to mixture C and allowed to react for 15 minutes; the light absorbance at 319 nm was then recorded.

Measurement of peroxynitrite anion ($ONOO^-$) scavenging activity

Peroxynitrite ($ONOO^-$) was generated through the method described by Beckman *et al.* [1] Specifically, an acidic solution (0.6 M HCl) of 5 mL H_2O_2 (0.7 M) was mixed with 5 mL KNO_2 (0.6 M) on an ice bath and then 5 mL of ice-cold NaOH (1.2 M) was added. Excess H_2O_2 was removed by treatment with MnO_2, and the reaction mixture was left overnight at −18°C. Peroxynitrite solution was collected from the top of the frozen mixture and the concentration was measured spectrophotometrically at 302 nm (ε = 1670 M^{-1} cm^{-1}). An Evans Blue bleaching assay was applied to measure peroxynitrite scavenging activity. The reaction mixture contained 0.01 M PBS (pH~7.4), DTPA (0.1 mM), NaCl (90 mM), KCl (5 mM), Evans Blue (12.5 μM), various doses of $H_{0.53}WO_3$ (10, 20, 30, 40 or 50 μg/mL) and peroxynitrite (1 mM) in a final volume of 1 mL. The mixture was reacted for 30 minutes, and the light absorbance at 600 nm was recorded.

Animal preparation

Male BALB/c mice (6–8-week-old) were purchased from GemPharmatech Co., Ltd. (China). All mice used in this work were housed in a specific pathogen-free facility and received care in compliance with the guidelines outlined in the Guide for the Care and Use of Laboratory Animals. Mice were maintained at 21 ± 1°C, in 40 % to 70 % humidity, and with a 12 h light-dark cycle (lights on from 8:00 to 20:00). All animal experiments were approved by the University of Science and Technology of China Animal Care and Use Committee (Approval ID: USTCACUC192401033).

Wound healing assessment in diabetic mice

The mice were randomly divided into three groups (n = 4 mice per group). To establish the diabetic mouse models, intraperitoneal injection of STZ (50 mg/kg) was administered for 5 consecutive days. Mice exhibiting fasting blood glucose levels higher than 16.7 mmol/L were included in this experiment. Full-thickness skin wounds with a diameter of 8 mm were then created on the backs of the mice using a dermatome. For each group, three different formulations (including PBS, WO_3, and H_xWO_3 powder were applied to the wounds). Photographs of the wounds were taken on days 0, 3, 7, and 12, with a ruler placed alongside the wounds as a scale bar for accurate area calculation. Wound closure was quantified using both Image FIJI software and caliper measurements, and the percentage of wound healing compared to day 0 was determined.

Histology and Immunohistochemistry

The mice were sacrificed, and their wound tissues were collected to examine the histological changes in the wound on day 7 and 12 after different treatments (n = 4 mice per group). Briefly, the skin tissues were fixed in 4% paraformaldehyde fixation solution, dehydrated, and embedded in paraffin. Sections of 5 μm thickness were prepared from the skin tissues using a microtome, and these sections were subsequently subjected to staining with H&E or Masson's trichrome staining to evaluate the histological characteristics of the wounds.

For the immunohistochemical studies, skin tissues were harvested and fixed with 4% paraformaldehyde, embedded in paraffin and then sectioned into 5 μm thick sections. Subsequently, the sections were dewaxed and rehydrated with xylene, ethanol, and deionized water. The slides were boiled for 90 seconds in 0.01 M sodium citrate buffer (pH 6.0) in pressure cooker for antigen retrieval, followed by blocking with 10% normal goat serum. For the evaluation of angiogenesis, primary antibodies against CD31 and α-SMA were utilized. CD206 and CD86 primary antibodies were used to determine macrophage phenotypes in the wound tissue. The expression of inflammatory cytokines, including IL-6, TNF-α, IL-10, and TGF-β, was assessed using

-

corresponding antibodies. Following incubation with the primary antibodies, the sections were treated with a horseradish peroxidase-conjugated secondary antibody for 1 hour at room temperature.

The sections were observed and photographed using the automated quantitative microscopy-based image analysis system TissueFAXS PLUS (TissueGnostics GmbH, Austria) with the 40 × objective. Granulation tissue and collagen fibers were quantified using Image FIJI software.

Biosafety analysis

The mice (n = 5 for each group) were sacrificed, and their wound tissues were collected on day 3 and 7 after different treatments to assess the biodegradability of H_xWO_3 using ICP-MS. Additionally, the hearts, livers, kidneys, lungs, and spleens were harvested and fixed in 4% PFA for subsequent H&E staining. The histological morphology of the tissues was examined using a light microscope (TissueFAXS PLUS). Blood parameters at preset time points after H_xWO_3 treatment were analyzed using the hematology analyzers (Sysmex, Japan).

Statistical analysis

Data are expressed as mean ± s.d. Statistical analysis between groups was performed using GraphPad Prism 8 or Origin 2022. For comparisons between two groups, unpaired two-tailed Student's t-tests were used. For data with multiple groups, statistically significant differences were assessed using one-way analysis of variance (ANOVA) or two-way ANOVA with Sidak's multiple comparisons test. The sample sizes (biological replicates), specific statistical tests, and the main effects of our statistical analyses for each experiment are detailed in each figure legend.

Materials Characterization

Powder X-ray diffraction (XRD) patterns were recorded using a Rigaku/Max-3A X-ray diffractometer with Cu Kα radiation (λ=1.54178 Å). Morphology and size of H_xWO_3 were investigated by scanning electron microscopy (Hitachi SU8220) at an operating voltage of 3.0 kV. The high-resolution transmission electron microscope (JEOL JEM2010) was operated at an accelerating voltage of 200 kV. Integrated differential phase contrast scanning transmission electron microscopy was performed on a Thermo Fisher Scientific Themis-Z operated at an accelerating voltage of 300 kV. Solid-state ^1H magic-angle-spinning (MAS) nuclear magnetic resonance (NMR) spectra were obtained with a 600 MHz NMR spectrometer (Bruker) at 300 K. UV-Vis light absorption spectra was measured on a Shimadzu spectrophotometer (UV-2600/2700). The concentration of W ions was determined by inductively coupled plasma mass spectrometry (ICP-MS) on an Optima 7300 DV (Perkin-Elmer Corporation).

DFT Calculation

All spin-polarized density functional theory (DFT) calculations were performed using the Perdew-Burke-Ernzerhofer (PBE) [2] functional in conjunction with the plane-wave projected augmented wave (PAW) [3] method as implemented in Vienna Ab Initial Simulation Package (VASP) [4,5]. To describe the strong Coulomb interaction of orbital electrons in the inner layers of W atoms, the DFT+U (U value of 6.2) calculations were performed following the approach suggested by Dudarev *et al.* [6] A Van der Waals (VDW) correction was performed to describe dispersion interactions within the system. The kinetic cutoff energy for the plane-wave basis set was 520 eV. The first Brillouin zone was sampled by a Monkhorst-Pack scheme with a 3 × 2 × 1 k-point grid for WO_3. To avoid spurious interactions between the system and its image due to the periodic boundary condition, we added a 15 Å vacuum space on the *c* axis. Structures were fully relaxed until the forces and energy converged to less than 0.02 eV/Å and 10^{-4} eV, respectively.

-

The Gibbs free energies during the reaction were calculated by the following formula:

$$G = E_{sol} + E_{ZPE} - TS$$

where E_{sol} is the electronic energy calculated using the VASPsol patch [7]; the zero-point energy E_{ZPE} and entropy S were obtained by calculating the frequency of the absorbed molecules; T is temperature (T = 298.15 K).

The large-scale atomistic simulation program (LASP) was applied for the first-principles transition state search. The structure of the transition state was verified by the unique imaginary frequency in the frequency calculation.

-

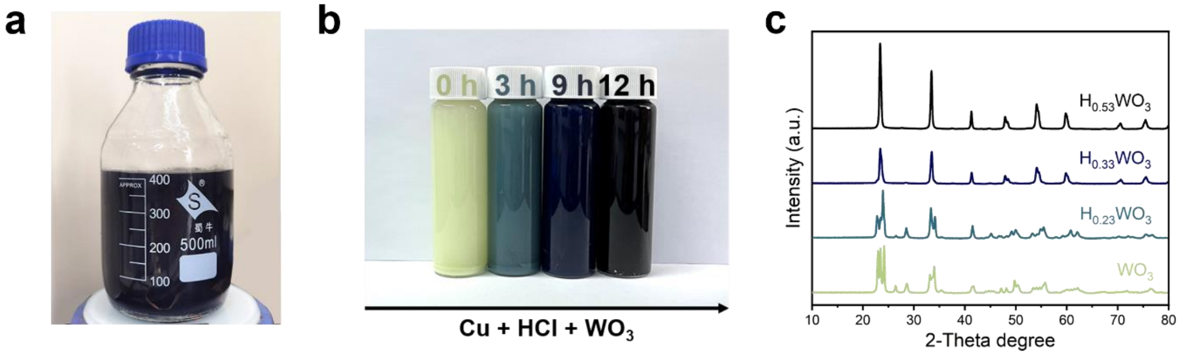

Figure S1. (a) Large-scale preparation of H_xWO_3 in a 500 mL glass bottle. (b) Sample photographs of pristine WO_3 and gradient-hydrogenated H_xWO_3 (from left to right). (c) XRD patterns of the H_xWO_3 samples at various H-doping time (from 0 h to 12 h).

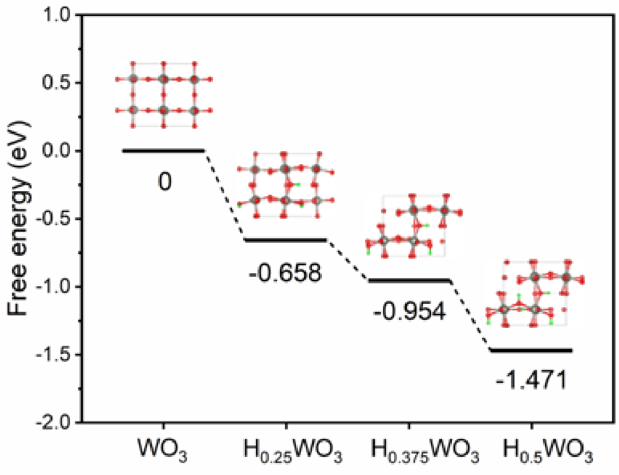

Figure S2. Gibbs free energy change of H_xWO_3 (values of x from 0 to 0.5) during hydrogen doping process, with each corresponding crystal structure depicted at the top (Green – hydrogen atoms).

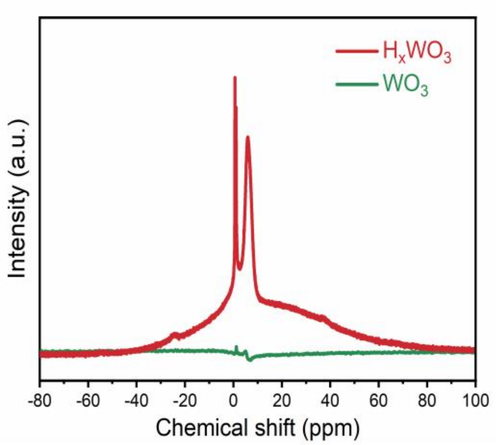

Figure S3. Solid-state ^{1}H NMR spectra of WO_3 and H_xWO_3.

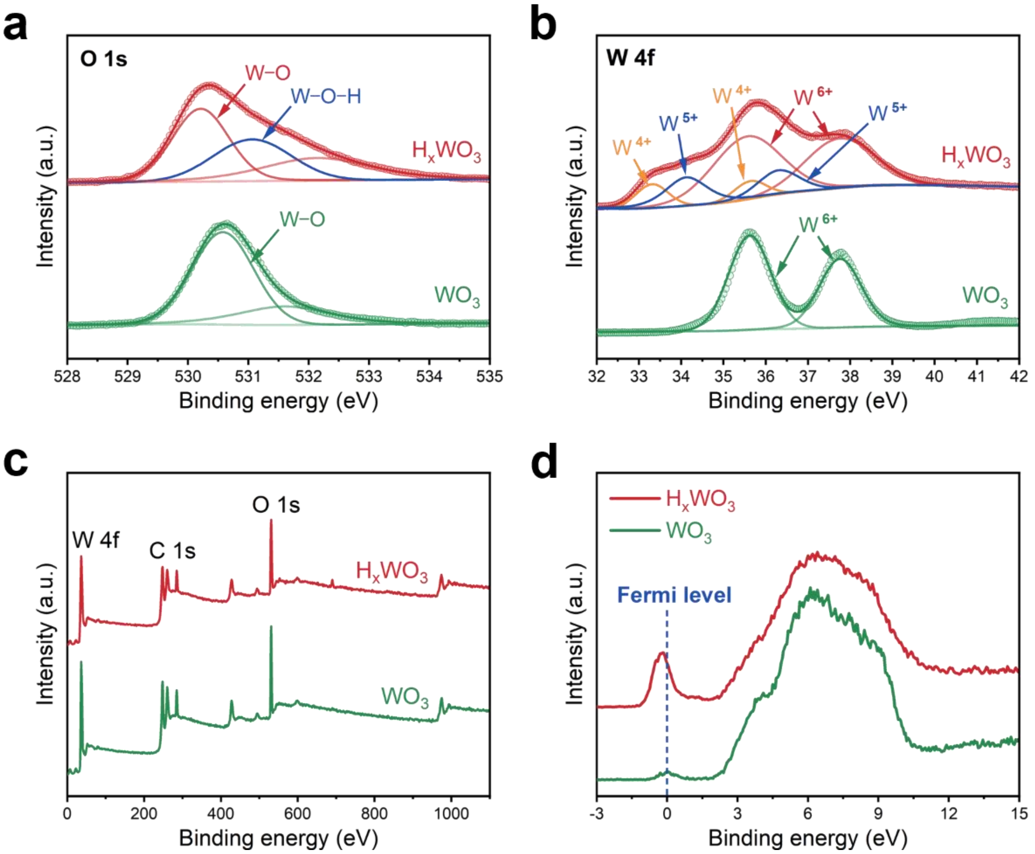

Figure S4. XPS at (a) O 1s and (b) the W 4f core levels of H_xWO_3 and WO_3. (c) Survey spectrum of H_xWO_3 and WO_3 identifying the presence of W and O elements. (d) VB-XPS spectra indicating the presence of excess free electrons near the Fermi level in H_xWO_3.

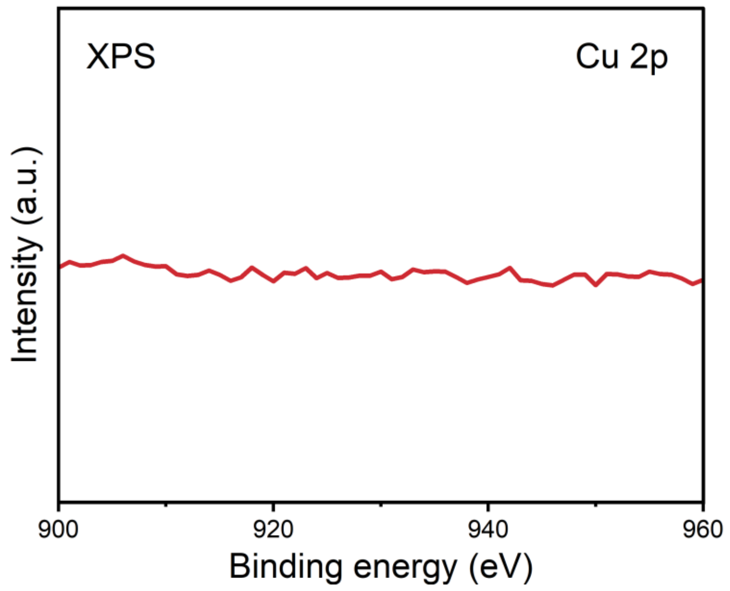

Figure S5. XPS spectrum of H_xWO_3 sample at Cu 2p region.

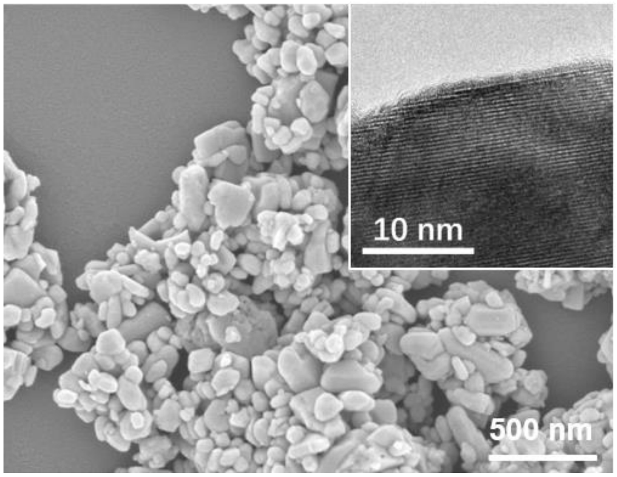

Figure S6. SEM image of pristine WO_3 nanoparticles; inset is HRTEM image of the crystalline surface.

-

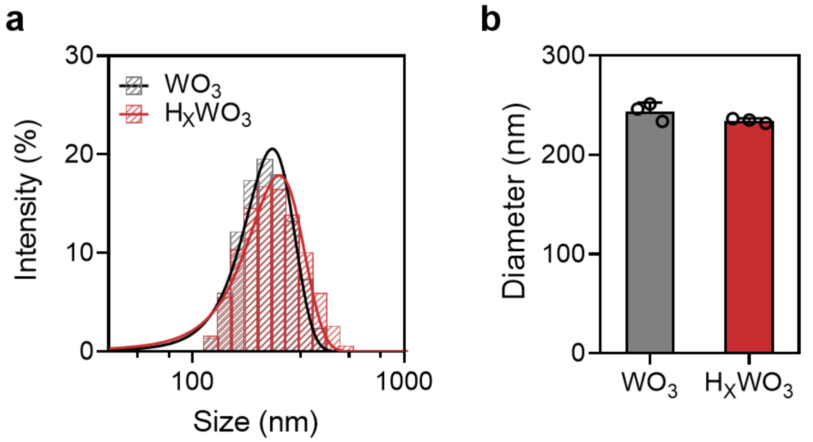

Figure S7. (a) Hydrodynamic size distributions and (b) measured average particle diameters of pristine WO_3 and H_xWO_3.

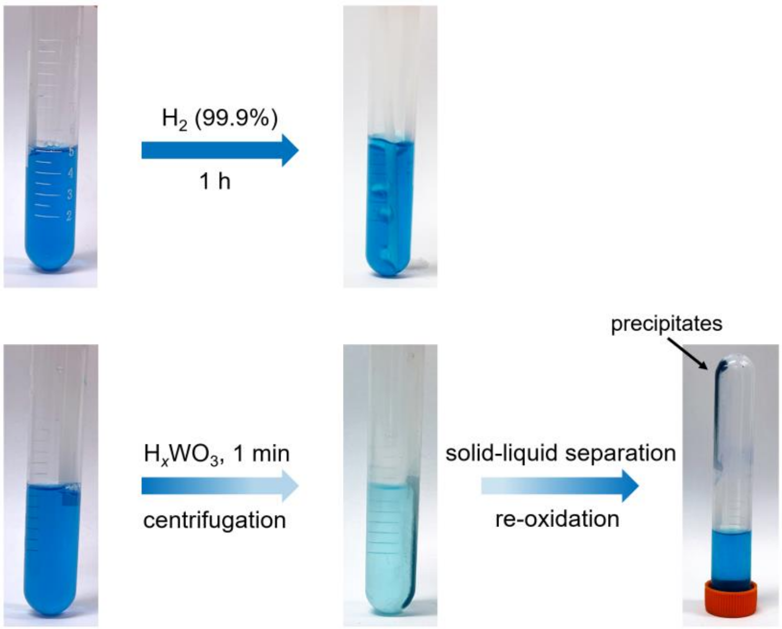

Figure S8. Experimental comparison of the reduction capabilities of atomic H and gaseous H_2.

-

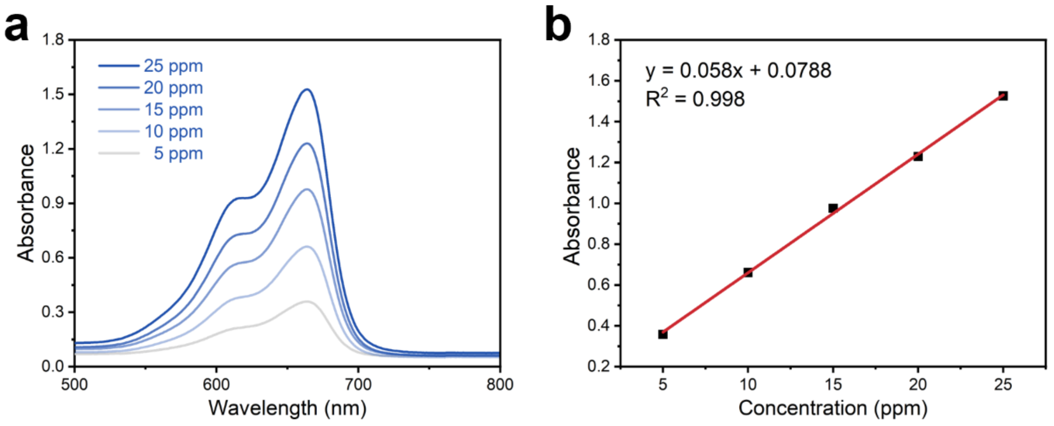

Figure S9. (a) UV-Vis absorbance spectra of methylene blue (MB) solutions at concentrations of 5, 10, 15, 20, and 25 ppm. (b) Linear calibration curve based on the peak absorbance at 663 nm.

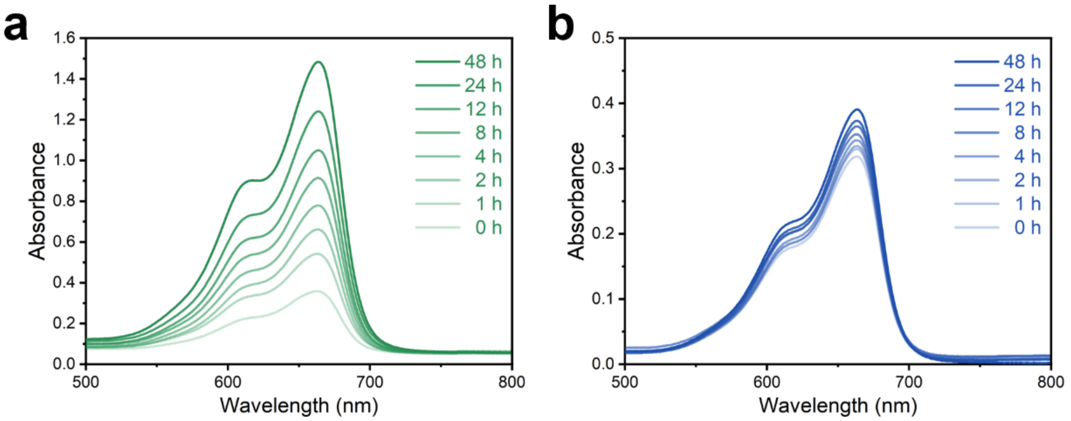

Figure S10. Dynamic evolution of the UV-Vis absorbance spectra of methylene blue (MB) solution for the determination of the amount of atomic hydrogen released from solid H_xWO_3 stored at (a) 37°C or (b) −18°C at different time intervals.

-

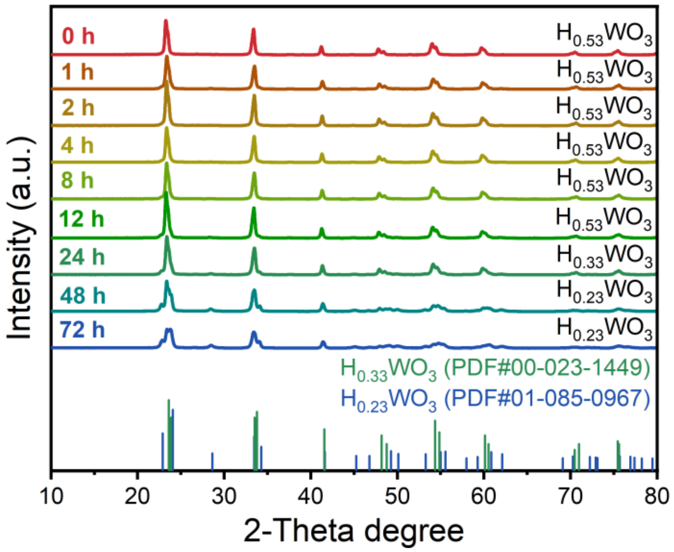

Figure S11. The ex-situ XRD patterns of H_xWO_3 during hydrogen release process at −18°C.

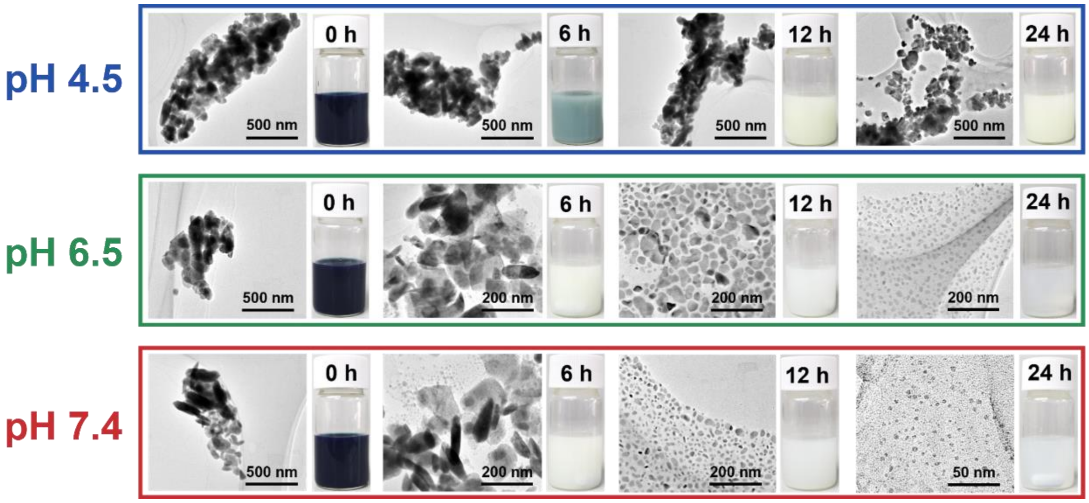

Figure S12. HRTEM images displaying the collapse process of H_xWO_3 during the pH-responsive degradation from 0 to 24 h.

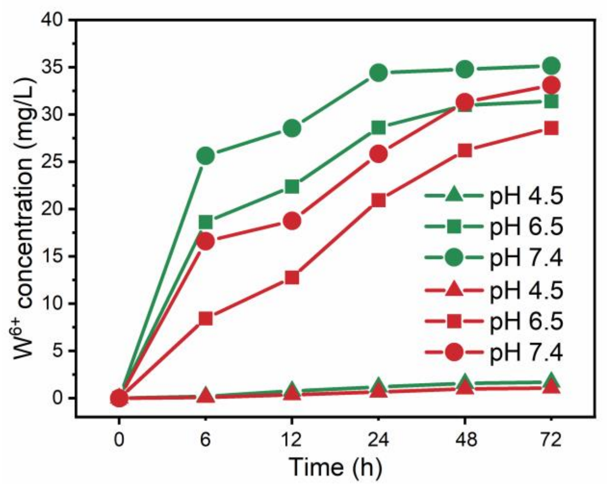

Figure S13. W ion concentrations measured by ICP-MS during degradation of WO_3 (green lines) and H_xWO_3 (red lines) in buffer solutions at different pH values (4.5, 6.5, and 7.4).

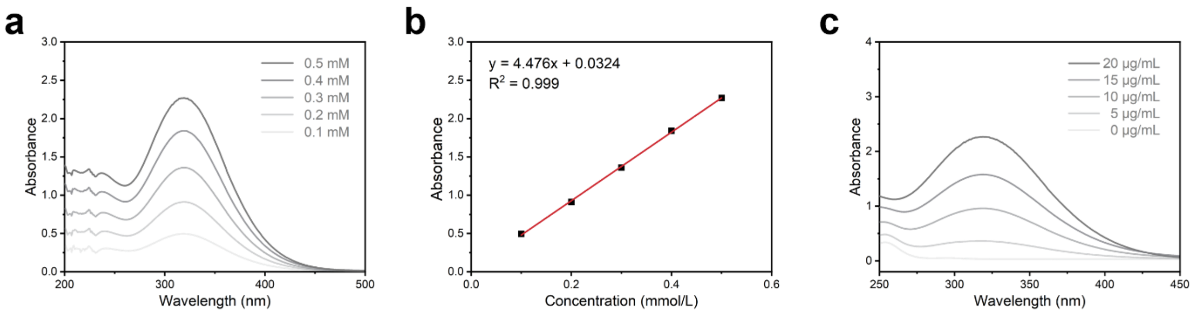

Figure S14. (a) UV-Vis absorbance spectra of $Ce(SO_4)_2$ solutions at concentrations of 0.1, 0.2, 0.3, 0.4, and 0.5 mmol/L. (b) Linear calibration curve based on the peak absorbance at 319 nm. (c) UV-Vis absorbance spectra of $Ce(SO_4)_2$ solution (0.5 mmol/L) treated with different concentrations of H_xWO_3.

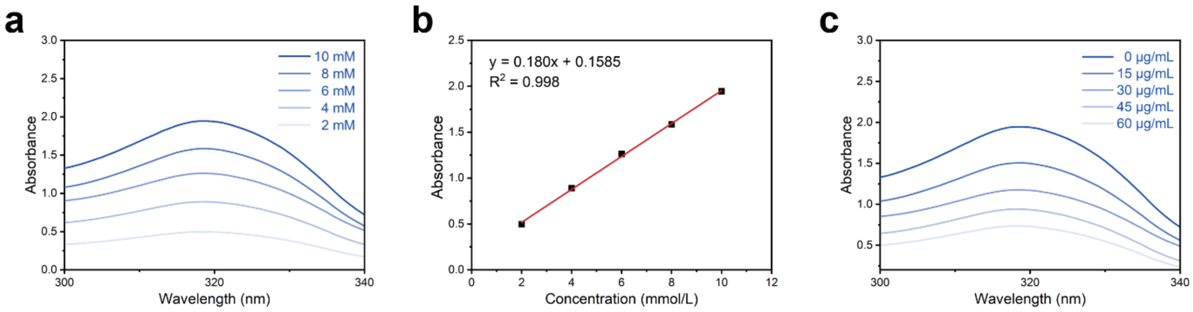

Figure S15. (a) UV-Vis absorbance spectra of pyrogallol solutions at concentrations of 2, 4, 6, 8, and 10 mmol/L. (b) Linear calibration curve based on the peak absorbance at 319 nm. (c) UV-Vis absorbance spectra of pyrogallol solution (10 mmol/L) treated with different concentrations of H_xWO_3.

-

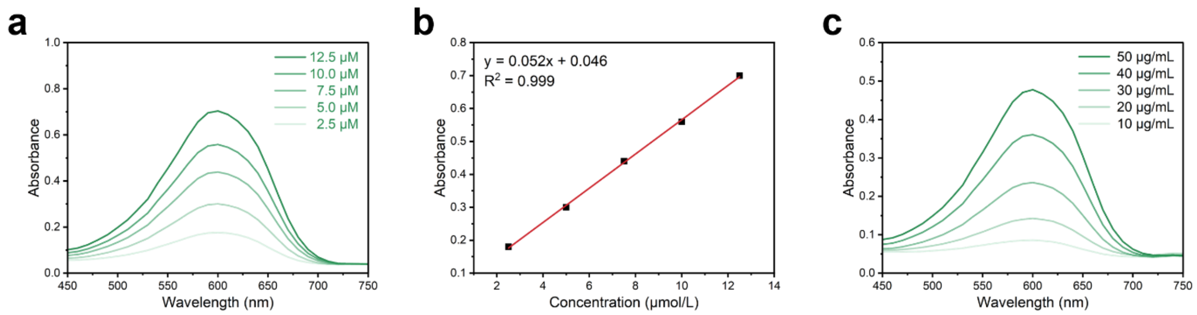

Figure S16. (a) UV-Vis absorbance spectra of Evans blue solutions at concentrations of 2.5, 5.0, 7.5, 10.0, and 12.5 μmol/L. (b) Linear calibration curve based on the peak absorbance at 600 nm. (c) UV-Vis absorbance spectra of Evans Blue solution (10 μmol/L) treated with different concentrations of H_xWO_3.

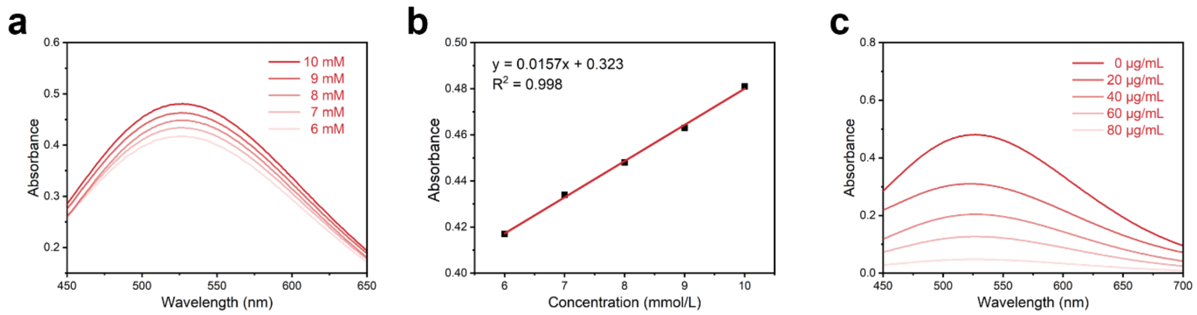

Figure S17. (a) UV-Vis absorbance spectra of salicylic acid (SA) solutions at concentrations of 6, 7, 8, 9, and 10 mmol/L. (b) Linear calibration curve based on the peak absorbance at 527 nm. (c) UV-Vis absorbance spectra of SA solution (10 mmol/L) treated with different concentrations of H_xWO_3.

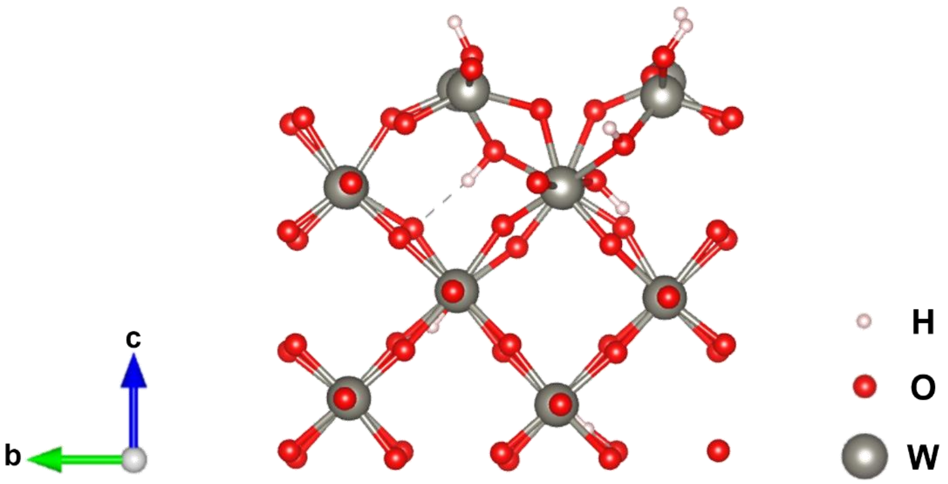

Figure S18. Atomic structure of $H_{0.5}WO_3$ (110) surface (side view).

-

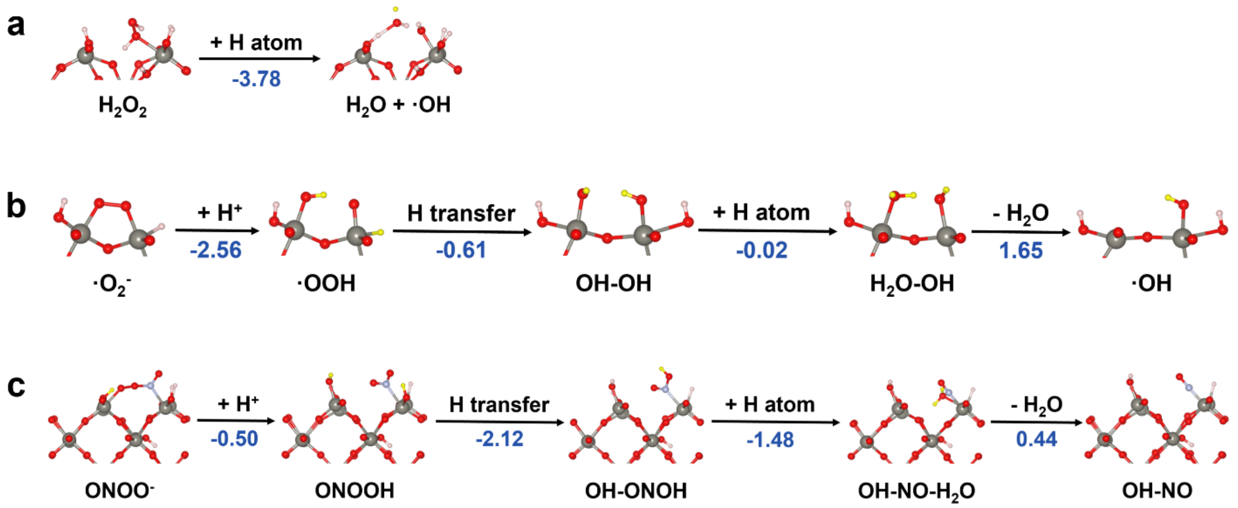

Figure S19. Schematic diagram of RONS scavenging pathways on $H_{0.5}WO_3$ (110) surface during reaction with (a) H_2O_2, (b) $•O_2^-$ and (c) $ONOO^-$. The blue number below the arrow represents the change in Gibbs free energy (eV) at each step.

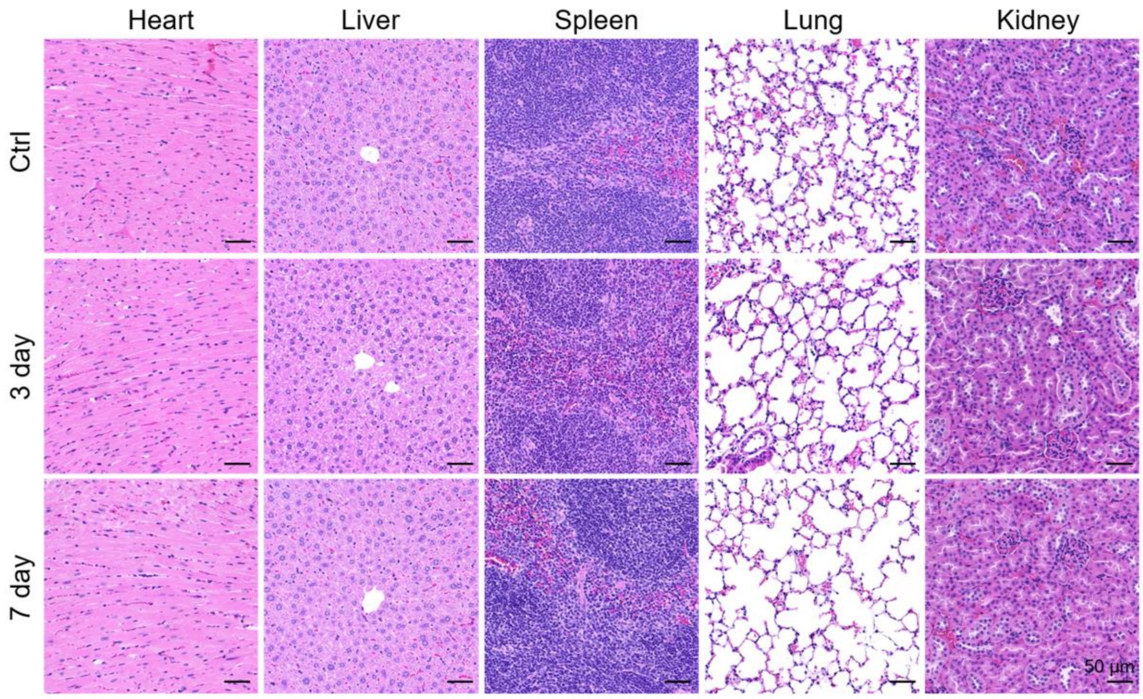

Figure S20. H&E-stained tissue sections of major organs at day 3 and 7 post H_xWO_3 treatment.

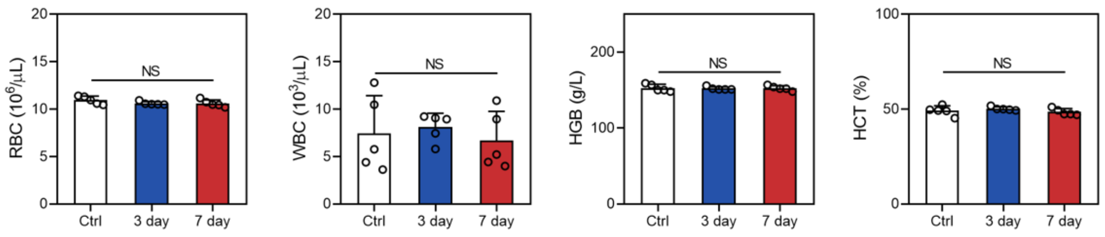

Figure S21. Blood parameters at various time points after *in vivo* applying H_xWO_3. Data are shown as mean ± s.d. (n=5). RBC, Red Blood Cell; WBC, White Blood Cell; HGB, Hemoglobin; HCT, Hematocrit.

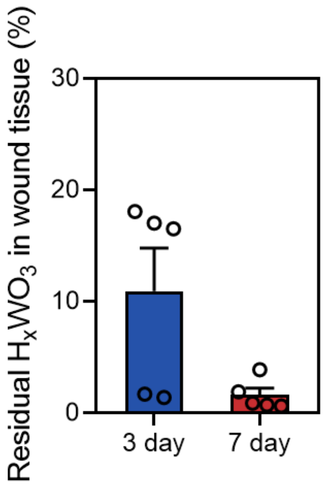

Figure S22. Residual H_xWO_3 content at day 3 and 7 in wound tissue (n = 5 mice per group).

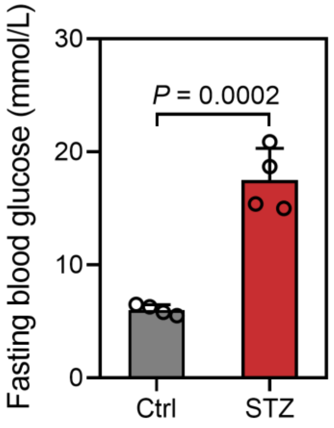

Figure S23. Fasting blood glucose levels of control and STZ-treatment group (n = 4 mice per group).

-

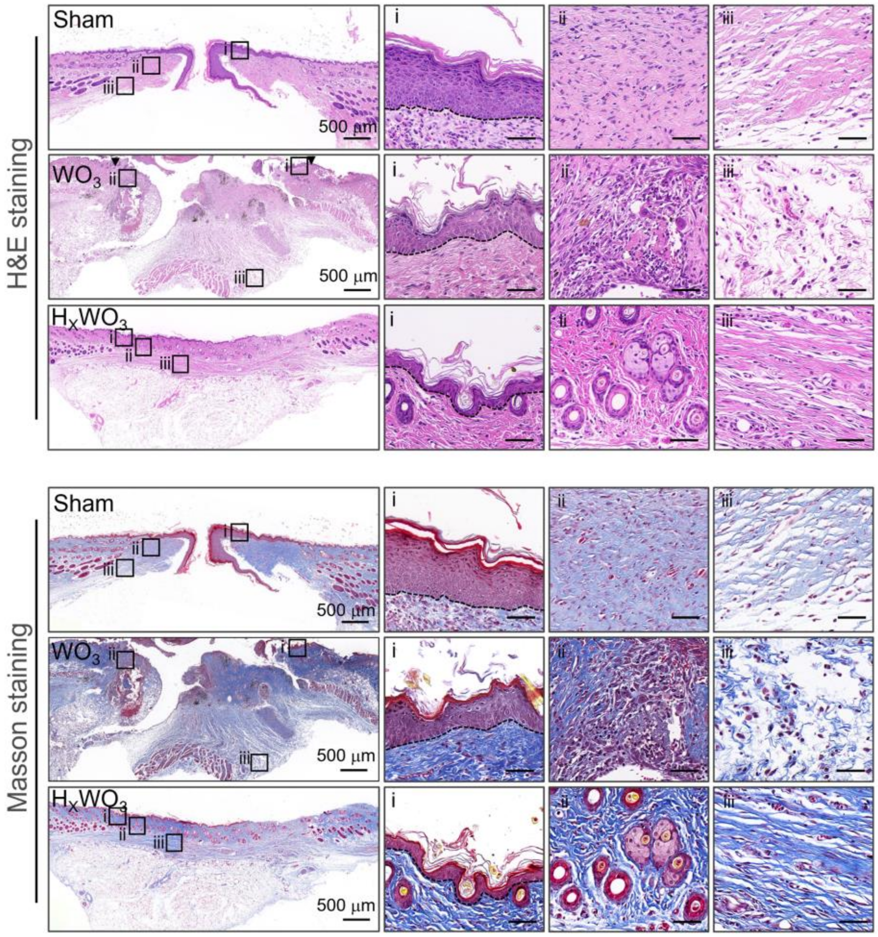

Figure S24. H&E staining images of the wound tissue on day 7, accompanied by local magnification of the epidermis (i), dermis (ii) and subcutaneous tissue (iii).

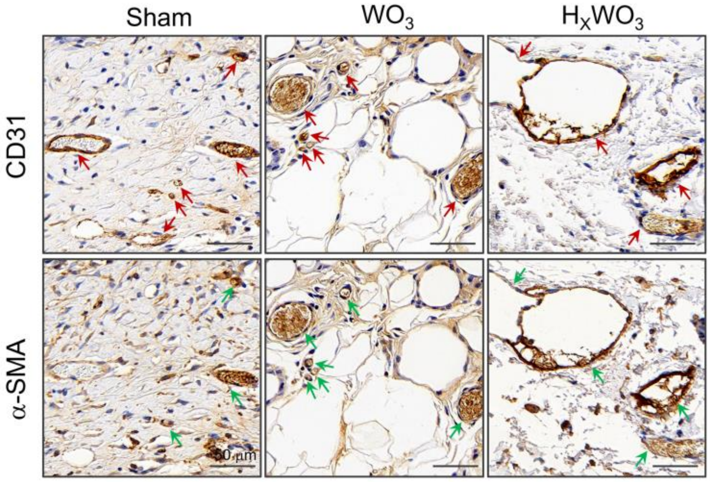

Figure S25. Representative images of CD31 and α-SMA immunostaining in wound tissue at day 12.

-

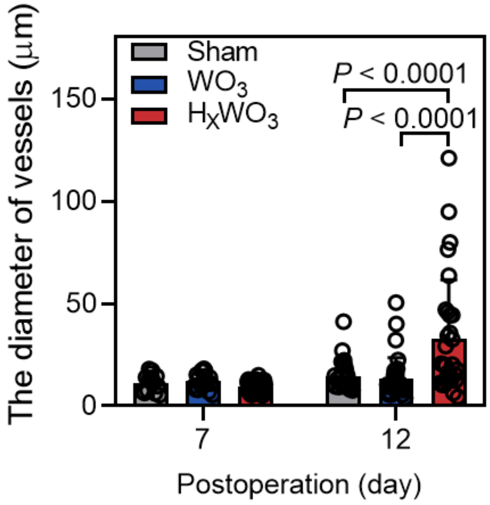

Figure S26. The diameter of vessels at day 7 and 12 after different treatments (n = 20 vessels from 4 biologically independent samples per group).

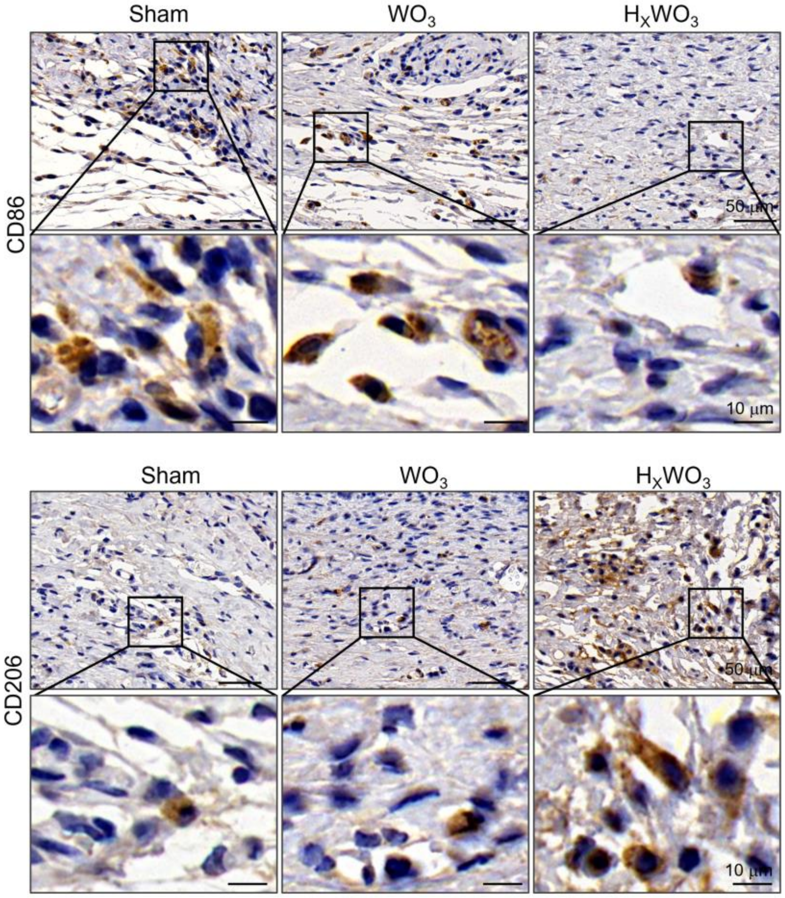

Figure S27. Representative images of CD86 and CD206 immunostaining in wound tissue on day 7.

-

Table S1. Comparison of reported therapeutic platforms with broad-spectrum RONS scavenging capabilities.

**Materials**	**Scavenging efficiency (%)**				**Bio-degradability**	**References**
	**Hydroxyl radical (·OH)**	**Hydrogen peroxide (H_2O_2)**	**Superoxide anion (·O_2^-)**	**peroxynitrite anion ($ONOO^-$)**		
**H_xWO_3**	80 μg/mL (92 %)	20 μg/mL (99 %)	60 μg/mL (63 %)	50 μg/mL (85 %)	Yes	This work
BL@B-SA_{50}	50 μg/mL (72 %)	100 μg/mL (50 %)	100 μg/mL (80 %)	N. A.	N. A.	*Nat. Nanotechnol.* **2023**, DOI 10.1038/s41565-023-01346-x.[8]
MN_x (M = Rh, Fe, Cu, V)	100 ng/mL (99 %)	517 U/μM (99 %)	11 μM (80 %)	N. A.	Yes	*Nat. Commun.* **2022**, *13*, 4744.[9]
Nanozyme hydrogel	N. A.	100 μL (99 %)	N. A.	N. A.	Yes	*Nat. Commun.* **2022**, *13*, 6758.[10]
PCZ@PB	N. A.	200 μM (90 %)	200 μM (80 %)	N. A.	Yes	*Nat. Commun.* **2022**, *13*, 6528.[11]
V_2C MXene	64 μg/mL (70 %)	20 μg/mL (30 %)	400 μg/mL (80 %)	N. A.	N. A.	*Nat. Commun.* **2021**, *12*, 2203.[12]
$Cu_{5.4}O$	150 ng/mL (80 %)	200 ng/mL (80 %)	200 ng/mL (80 %)	N. A.	Yes	*Nat. Commun.* **2020**, *11*, 2788.[13]
Pt@PCN222-Mn	N. A.	4 μg/mL (50 %)	80 μg/mL (82 %)	N. A.	N. A.	*Sci. Adv.* **2020**, *6*, eabb2695.[14]
CeO_2@ZIF-8	15 μg/mL (45 %)	N. A.	20 μg/mL (60 %)	N. A.	Yes	*Sci. Adv.* **2020**, *6*, eaay9751.[15]
H-silicene	100 μg/mL (99 %)	N. A.	100 μg/mL (99 %)	N. A.	Yes	*J. Am. Chem. Soc.* **2022**, *144*, 14195.[16]
$LiMn_2O_4$	200 μg/mL (70 %)	200 μg/mL (70 %)	50 μg/mL (50 %)	N. A.	Yes	*Angew. Chemie Int. Ed.* **2022**, *61*, e202201101.[17]
Co/PMCS	10 μM (97 %)	5 μM (99 %)	20 μM (90 %)	80 μM (80 %)	Yes	*Angew. Chemie Int. Ed.* **2020**, *59*, 5108.[18]

-

Mn-Co_3O_4	10 μg/mL (60 %)	50 μg/mL (85 %)	10 μg/mL (60 %)	N. A.	Yes	*Adv. Mater.* **2022**, *34*, 2207275.[19]
TMSN@PM	125 μg/mL (76 %)	125 μg/mL (80 %)	125 μg/mL (78 %)	N. A.	Yes	*Adv. Mater.* **2022**, *34*, 2109004.[20]
APBP	N. A.	0.1 mM (80 %)	N. A.	N. A.	Yes	*Adv. Mater.* **2021**, *33*, 2100746.[21]
Antimicrobial peptides	100 μg/mL (98 %)	N. A.	100 μg/mL (84 %)	N. A.	Yes	*Adv. Funct. Mater.* **2023**, 2214454.[22]
CeO_2 NWs	5 μg/mL (81 %)	N. A.	2 μg/mL (98 %)	N. A.	N. A.	*Adv. Funct. Mater.* **2023**, 2300013.[23]
Fe_2NC@Se	10 μg/mL (60 %)	500 μg/mL (83 %)	100 μg/mL (90 %)	N. A.	N. A.	*Adv. Funct. Mater.* **2022**, 2204025.[24]
$PdH_{0.12}$	N. A.	200 μg/mL (80 %)	200 μg/mL (50 %)	N. A.	N. A.	*Adv. Funct. Mater.* **2021**, *31*, 2104892.[25]
$NbSe_2$ nanosheets	160 μg/mL (total free radical removal 95 %)			160 μg/mL (67 %)	Yes	*Adv. Funct. Mater.* **2020**, *30*, 2001593.[26]
Tetrapod PdH nanozyme	100 μg/mL (75 %)	150 μg/mL (65 %)	125 μg/mL (65 %)	N. A.	N. A.	*ACS Nano* **2022**, *16*, 15959.[27]
SeNG	N. A.	10 mg/mL (40 %)	N. A.	N. A.	N. A.	*ACS Nano* **2022**, *16*, 13037.[28]
Pt/CeO_2	375 μg/mL (≈100 %)	50 μg/mL (77.34 %)	10 μg/mL (≈50 %)	375 μg/mL (86 %)	N.A.	*ACS Nano* **2019**, *13*, 11552.[29]
Ultrasmall rhodium	80 μg/mL (<60 %)	N. A.	80 μg/mL (93 %)	80 μg/mL (≈85 %)	N. A.	*Nano Lett.* **2020**, *20*, 3079.[30]
Carbogenic nanozyme	30 μM (>80 %)	N. A.	21 μM (>90 %)	42 μM (≈85%)	N.A.	*Nano Lett.* **2019**, *19*, 4527.[31]
Hollow Prussian blue	(62 %)	(88 %)	(79 %)	30 μg/mL (72 %)	N. A.	*Nano Lett.* **2019**, *19*, 2812.[32]

BL@B-SA_{50}: artificial-enzyme-modified (Fe SA) *Bifidobacterium longum* probiotics.

PCZ@PB: platelet membrane-coated ceria-zoledronic acid nanocomposites with probucol.

Co/PMCS: nitrogen-doped carbon-supported atomically dispersed Co-porphyrin centers.

-

TMSN@PM: composed of platelet membrane (PM)-coated, tempol-grafted, manganese-doped, mesoporous silica nanoparticles (MSN).

APBP: ROS-responsive dendrimer-peptide conjugate.

SeNG: diselenide-bridged hyaluronic acid nanogel.

-

REFERENCES

1. Beckman JS, Beckman TW and Chen J *et al.* Apparent hydroxyl radical production by peroxynitrite: implications for endothelial injury from nitric oxide and superoxide. *Proc Natl Acad Sci* 1990; **87**: 1620–4.
2. Perdew JP, Burke K and Ernzerhof M. Generalized gradient approximation made simple. *Phys Rev Lett* 1997; **78**: 1396–1396.
3. Blöchl PE. Projector augmented-wave method. *Phys Rev B* 1994; **50**: 17953–79.
4. Kresse G and Furthmüller J. Efficient iterative schemes for ab initio total-energy calculations using a plane-wave basis set. *Phys Rev B* 1996; **54**: 11169–86.
5. Kresse G and Joubert D. From ultrasoft pseudopotentials to the projector augmented-wave method. *Phys Rev B* 1999; **59**: 1758–75.
6. Dudarev SL, Botton GA and Savrasov SY *et al.* Electron-energy-loss spectra and the structural stability of nickel oxide: an LSDA+U study. *Phys Rev B* 1998; **57**: 1505–9.
7. Mathew K, Sundararaman R and Letchworth-Weaver K *et al.* Implicit solvation model for density-functional study of nanocrystal surfaces and reaction pathways. *J Chem Phys* 2014; **140**: 084106.
8. Cao F, Jin L and Gao Y *et al.* Artificial-enzymes-armed Bifidobacterium longum probiotics for alleviating intestinal inflammation and microbiota dysbiosis. *Nat Nanotechnol* 2023; doi: 10.1038/s41565-023-01346-x.
9. Zhang S, Li Y and Sun S *et al.* Single-atom nanozymes catalytically surpassing naturally occurring enzymes as sustained stitching for brain trauma. *Nat Commun* 2022; **13**: 4744.
10. Zhao Y, Song S and Wang D *et al.* Nanozyme-reinforced hydrogel as a H_2O_2-driven oxygenerator for enhancing prosthetic interface osseointegration in rheumatoid arthritis therapy. *Nat Commun* 2022; **13**: 6758.
11. Fu X, Yu X and Jiang J *et al.* Small molecule-assisted assembly of multifunctional ceria nanozymes for synergistic treatment of atherosclerosis. *Nat Commun* 2022; **13**: 6528.
12. Feng W, Han X and Hu H *et al.* 2D vanadium carbide MXenzyme to alleviate ROS-mediated inflammatory and neurodegenerative diseases. *Nat Commun* 2021; **12**: 2203.
13. Liu T, Xiao B and Xiang F *et al.* Ultrasmall copper-based nanoparticles for reactive oxygen species scavenging and alleviation of inflammation related diseases. *Nat Commun* 2020; **11**: 2788.
14. Liu Y, Cheng Y and Zhang H *et al.* Integrated cascade nanozyme catalyzes *in vivo* ROS scavenging for anti-inflammatory therapy. *Sci Adv* 2020; **6**, eabb2695.
15. He L, Huang G and Liu H *et al.* Highly bioactive zeolitic imidazolate framework-8-capped nanotherapeutics for efficient reversal of reperfusion-induced injury in ischemic stroke. *Sci Adv* 2020; **6**: eaay9751.
16. You Y, Zhu Y-X and Jiang J *et al.* Water-enabled H_2 generation from hydrogenated silicon nanosheets for efficient anti-inflammation. *J Am Chem Soc* 2022; **144**: 14195–206.
17. Wang Q, Cheng C and Zhao S *et al.* A valence ‐ engineered self ‐ cascading antioxidant nanozyme for the therapy of inflammatory bowel disease. *Angew Chemie Int Ed* 2022; **61**, e202201101.
18. Cao F, Zhang L and You Y *et al.* An enzyme-mimicking single-atom catalyst as an efficient multiple reactive oxygen and nitrogen species scavenger for sepsis management. *Angew Chemie Int Ed* 2020; **59**: 5108–15.
19. Tian Q, Wang W and Cao L *et al.* Multifaceted catalytic ROS ‐ scavenging via electronic modulated metal oxides for regulating stem cell fate. *Adv Mater* 2022; **34**: 2207275.
20. Li X, Liu Y and Qi X *et al.* Sensitive activatable nanoprobes for real ‐ time ratiometric magnetic resonance imaging of reactive oxygen species and ameliorating inflammation *in vivo*. *Adv Mater* 2022; **34**: 2109004.

-

21. Liu P, Zhang T and Chen Q *et al.* Biomimetic dendrimer–peptide conjugates for early multi‐target therapy of Alzheimer's disease by inflammatory microenvironment modulation. *Adv Mater* 2021; **33**: 2100746.
22. Teng R, Yang Yand Zhang Z *et al.* In situ enzyme‐induced self‐assembly of antimicrobial‐antioxidative peptides to promote wound healing. *Adv Funct Mater* 2023: 2214454.
23. Liu Q, Shi G and Yang L *et al.* Length‐controlled construction of ceria nanowires with ultrafine diameter and stable morphology for targeted acute lung injury therapy. *Adv Funct Mater* 2023: 2300013.
24. Tian R, Ma H and Ye W *et al.* Se‐containing MOF coated dual‐Fe‐atom nanozymes with multi‐enzyme cascade activities protect against cerebral ischemic reperfusion injury. *Adv Funct Mater* 2022; **2204025**: 2204025.
25. Xu M, Zhou Y and Ren C *et al.* Palladium hydride nanopocket cubes and their H_2‐therapy function in amplifying inhibition of foam cells to attenuate atherosclerosis. *Adv Funct Mater* 2021; **31**: 2104892.
26. Miao Z, Huang D and Wang Y *et al.* Safe‐by‐design exfoliation of niobium diselenide atomic crystals as a theory‐oriented 2D nanoagent from anti‐inflammation to antitumor. *Adv Funct Mater* 2020; **30**: 2001593.
27. Hu R, Dai C and Dong C *et al.* Living macrophage-delivered tetrapod PdH nanoenzyme for targeted atherosclerosis management by ROS scavenging, hydrogen anti-inflammation, and autophagy activation. *ACS Nano* 2022; **16**: 15959–76.
28. Xu J, Chu T and Yu T *et al.* Design of diselenide-bridged hyaluronic acid nano-antioxidant for efficient ROS scavenging to relieve colitis. *ACS Nano* 2022; **16**: 13037–48.
29. Yan R, Sun S and Yang J *et al.* Nanozyme-based bandage with single-atom catalysis for brain trauma. *ACS Nano* 2019; **13**: 11552–60.
30. Miao Z, Jiang S and Ding M *et al.* Ultrasmall rhodium nanozyme with RONS scavenging and photothermal activities for anti-inflammation and antitumor theranostics of colon diseases. *Nano Lett* 2020; **20**: 3079–89.
31. Mu X, He H and Wang J *et al.* Carbogenic nanozyme with ultrahigh reactive nitrogen species selectivity for traumatic brain injury. *Nano Lett* 2019; **19**: 4527–34.
32. Zhang K, Tu M and Gao W *et al.* Hollow prussian blue nanozymes drive neuroprotection against ischemic stroke via attenuating oxidative stress, counteracting inflammation, and suppressing cell apoptosis. *Nano Lett* 2019; **19**: 2812–23.
